# Evaluating the Adoption of mHealth Technologies by Community Health Workers to Improve the Use of Maternal Health Services in Sub-Saharan Africa: Systematic Review

**DOI:** 10.2196/55819

**Published:** 2024-09-24

**Authors:** Chiyembekezo Kachimanga, Haules Robbins Zaniku, Titus Henry Divala, Johannes C.F Ket, Joia S Mukherjee, Daniel Palazuelos, Alexandra V Kulinkina, Ibukun-Oluwa Omolade Abejirinde, Thomas van den Akker

**Affiliations:** 1 Athena Institute Vrije Universiteit Amsterdam Amsterdam Netherlands; 2 Kamuzu University of Health Sciences Blantyre Malawi; 3 Ministry of Health Neno Malawi; 4 Medical Library Vrije Universiteit Amsterdam Amsterdam Netherlands; 5 Partners In Health Boston, MA United States; 6 Swiss Tropical and Public Health Institute Allschwil Switzerland; 7 University of Basel Basel Switzerland; 8 Women College Hospital Institute for Health System Solutions and Virtual Care Toronto, ON Canada; 9 Dalla Lana School of Public Health University of Toronto Toronto, ON Canada; 10 Department of Obstetrics and Gynaecology Leiden University Medical Center Leiden Netherlands

**Keywords:** maternal health, antenatal care, postnatal care, facility-based births, sub-Saharan Africa, mobile health, mHealth, review, narrative synthesis, mobile phone

## Abstract

**Background:**

Limited information exists on the impact of mobile health (mHealth) use by community health workers (CHWs) on improving the use of maternal health services in sub-Saharan Africa (SSA).

**Objective:**

This systematic review addresses 2 objectives: evaluating the impact of mHealth use by CHWs on antenatal care (ANC) use, facility-based births, and postnatal care (PNC) use in SSA; and identifying facilitators and barriers to mHealth use by CHWs in programs designed to increase ANC use, facility-based births, and PNC use in SSA using a sociotechnical system approach.

**Methods:**

We searched for articles in 6 databases (MEDLINE, CINAHL, Web of Science, Embase, Scopus, and Africa Index Medicus) from inception up to September 2022, with additional articles identified from Google Scholar. After article selection, 2 independent reviewers performed title and abstract screening, full-text screening, and data extraction using Covidence software (Veritas Health Innovation Ltd). In addition, we manually screened the references lists of the included articles. Finally, we performed a narrative synthesis of the outcomes.

**Results:**

Among the 2594 records retrieved, 10 (0.39%) studies (n=22, 0.85% articles) met the inclusion criteria and underwent data extraction. The studies were published between 2012 and 2022 in 6 countries. Of the studies reporting on ANC outcomes, 43% (3/7) reported that mHealth use by CHWs increased ANC use. Similarly, of the studies reporting on facility-based births, 89% (8/9) demonstrated an increase due to mHealth use by CHWs. In addition, in the PNC studies, 75% (3/4) showed increased PNC use associated with mHealth use by CHWs. Many of the studies reported on the importance of addressing factors related to the social environment of mHealth-enabled CHWs, including the perception of CHWs by the community, trust, relationships, digital literacy, training, mentorship and supervision, skills, CHW program ownership, and the provision of incentives. Very few studies reported on how program goals and culture influenced mHealth use by CHWs. Providing free equipment, accessories, and internet connectivity while addressing ongoing challenges with connectivity, power, the ease of using mHealth software, and equipment maintenance support allowed mHealth-enabled CHW programs to thrive.

**Conclusions:**

mHealth use by CHWs was associated with an increase in ANC use, facility-based births, and PNC use in SSA. Identifying and addressing social and technical barriers to the use of mHealth is essential to ensure the success of mHealth programs.

**Trial Registration:**

PROSPERO CRD42022346364; https://www.crd.york.ac.uk/prospero/display_record.php?RecordID=346364

## Introduction

### Background

Sub-Saharan Africa (SSA) continues to have the highest maternal morbidity and mortality globally [1,2]. The region contributes only 15% of the world’s population [3] while accounting for 70% of all maternal deaths [4]. In 2020, SSA had a maternal mortality ratio (MMR) of 545 maternal deaths per 100,000 live births and a 1 in 40 lifetime risk of maternal death [5]. These estimates are significantly higher than in any other region of the world [5].

Between 2000 and 2015, the MMR decreased in many regions, including SSA [6]. Unfortunately, recent trends in the MMR have not been promising; it has either increased or remained the same between 2016 and 2020 [7,8]. There are projections that the MMR may stay the same or increase by 2030 [9]. As such, there is a need for innovative approaches to reduce the MMR.

One way to accelerate the reduction in the MMR is to improve the coverage of maternal health services [7]. This includes improving the use of maternal health services within the continuum of care, that is, ≥4 antenatal care (ANC) contacts, facility-based births attended by skilled attendants, and early postnatal care (PNC) [7]. Providing high-quality care along the maternal health continuum has been shown to reduce maternal mortality [10-14]. However, the use of available services remains a significant challenge in SSA; for example, the use of ANC and PNC among women of reproductive ages between 15 to 24 years in 28 SSA countries was only 55% and 40%, respectively [15]. Studies in SSA showed lower rates of facility-based births, with women in rural areas having lower rates of facility-based births than those in urban areas [16,17]. Using data from the most recent surveys in SSA countries, Wan et al [18] and Straneo et al [19] found that only 7 out of 10 pregnant women give birth in health facilities in the region.

The impact of community health workers (CHWs) on increasing the use of maternal health services has been established [20]. Working collaboratively with communities, health facilities, national ministries of health (MOHs), and international health agencies, CHWs advocate for improved care and reduce cultural and other barriers preventing women from accessing maternal health services [21]. In addition, CHWs provide education, identify and refer women seeking maternal health services to health facilities, and may offer case management for selected health conditions [22]. Therefore, CHWs are an essential component in reducing maternal deaths, increasing the use of maternal health services, and eventually achieving the United Nations’ Sustainable Development Goal 3.

To improve the efficiency of the tasks and responsibilities carried out by CHWs and help support improvements in clinical outcomes, mobile health (mHealth) technologies are increasingly being introduced to CHW programs [23]. mHealth is the use of mobile and wireless technologies in health care [24]. In general, evidence has shown that mHealth can improve outcomes in patients with chronic diseases, tuberculosis, and HIV infection [25]. In maternal health, mHealth has been shown to improve the coverage of ANC, facility-based births, and PNC [26,27]. However, previous reviews have not specifically examined the impact of mHealth tools when used by CHWs as the primary implementers. Specifically for CHWs, mHealth has been used to train them, improve their performance and retention, support data collection, support patient adherence to medication, and provide clinical decision support [28-32]. There is no review on mHealth use by CHWs to improve the maternal health continuum of care in SSA.

### Objectives

Although some of the results in the aforementioned studies hold promise regarding the use of mHealth by CHWs in general, there is a lack of robust evidence on the impact of mHealth use by CHWs to improve the use of services along the maternal health continuum of care in SSA, especially when compared with CHWs who do not use mHealth. This review aims to provide evidence synthesis on the impact of mHealth use by CHWs in SSA to improve the coverage of maternal health services in comparison with CHWs not using mHealth. In addition, it examines the factors that support or hinder the successful implementation of mHealth for the improved use of maternal health services. These aims are captured in 2 objectives. First, we assessed the impact of mHealth use by CHWs on ANC use, facility-based births, and PNC use in SSA, comparing the outcomes with those achieved by CHWs not using mHealth. Second, we reviewed the facilitators and barriers to mHealth use by CHWs in programs designed to increase ANC use, facility-based births, and PNC use.

## Methods

### Overview

This systematic review adheres to the PRISMA (Preferred Reporting Items for Systematic Reviews and Meta-Analyses) checklist (Multimedia Appendix 1) and guidelines [33]. As this is a mixed methods systematic review, we synthesized and integrated findings from both quantitative and qualitative studies to provide a more comprehensive understanding of the research question. We registered the review with PROSPERO (CRD42022346364), and the protocol was published previously [34].

### Eligibility Criteria

The review included studies involving eligible women of reproductive age (15-49 y) using care across the maternal health continuum: pregnant women using ANC at any gestational age, pregnant women accessing intrapartum care at health care facilities, and women accessing PNC up to 42 days after giving birth regardless of the mode of giving birth. We included all studies that reported the use of mHealth by CHWs to improve the use of these 3 services. CHWs were included if they met the definition set by the World Health Organization (WHO): “health workers based in communities...who are either paid or volunteer, who are not professionals, and who have fewer than 2 years training but at least some training” [35]. For intervention studies included in the review, the comparator was CHW programs that were not using mHealth. We included ANC visits, facility-based births, and PNC visits as outcomes. We also collected qualitative data about facilitators and barriers to mHealth use by CHWs as described in the included studies. Further details and review criteria are outlined in the published protocol [34].

### Search Strategy and Data Sources

We searched 6 databases (Scopus, MEDLINE, CINAHL, Web of Science, Embase, and Africa Index Medicus) from inception up to September 2022. To develop the concepts for search terms, we combined the following concepts: “women accessing maternal health services (pregnancy OR childbirth OR postnatal care) AND mHealth AND community health workers AND SSA countries.” We included all known synonyms and related terms identified from the literature. We adapted the search terms to each database. Due to challenges in reproducibility, we used Google Scholar as part of reference checking [34]. For Google Scholar, we developed search terms mirroring these major concepts and searched the first 1000 results for any new articles that met the inclusion criteria but were not captured by the other databases. The search strategy, including search terms for Google Scholar, is presented in Multimedia Appendix 2. We included randomized controlled trials (RCTs), quasi-experimental studies, nonexperimental quantitative studies, qualitative studies, and mixed methods studies that met the inclusion criteria. We excluded reviews and other summary-type articles, policy documents, commentaries, abstracts and conference proceedings, case reports, and protocols. Manual searches were conducted by reviewing the references lists of systematic reviews and all included articles. The included articles were limited to the SSA region as defined by the World Bank [36]. To reduce language bias, ensure the identification of all relevant studies regardless of publication time, and allow the generalization of findings to SSA, we did not limit the search by language or year of publication.

### Data Extraction

JCFK retrieved all studies from the electronic databases. CK and HRZ independently screened the titles and abstracts of the extracted articles, independently assessed full-text articles for inclusion, and conducted manual searches. An audit log was kept throughout the process, including documentation of reasons for exclusion. CK and HRZ performed the data extraction. TvdA resolved all discrepancies. Covidence software (Veritas Health Innovation Ltd) was used for the screening and data extraction [37].

We extracted information on the authors; publication year; study designs; country and geographic scope; the type and scope of work performed by CHWs; and mHealth characteristics, including platforms used, devices used, delivery methods, and the functions of mHealth. We also extracted the study results based on the outcomes as well as the facilitators and barriers to mHealth use.

### Risk-of-Bias Assessment, Analysis, and Synthesis

All included studies underwent a risk-of-bias assessment conducted by CK and HRZ using the Mixed Methods Assessment Tool [38]. Regardless of the results of the risk-of-bias assessment, we included all studies in data extraction, analysis, and synthesis. Due to the heterogeneity of the studies, we conducted a narrative synthesis using the 3 steps proposed by Popay et al [39]. Narrative synthesis involves synthesizing data using words and text, rather than statistical methods that are often used for qualitative studies or when meta-analysis is not possible due to heterogeneity in the study designs or outcomes.

**Figure 1:**
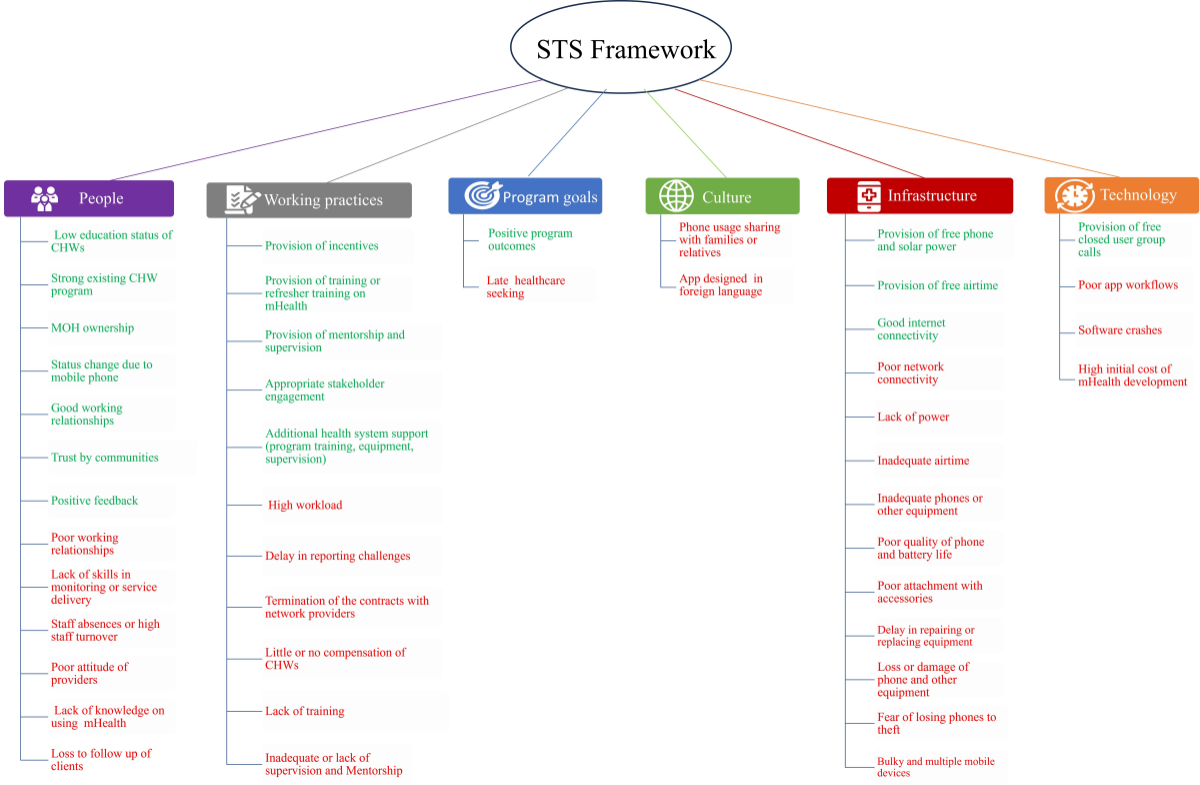
Facilitators and barriers mapped according to the sociotechnical system (STS) framework. mHealth: mobile health.

First, a preliminary synthesis based on the review objectives was performed. For the impact of mHealth use by CHWs on the use of maternal health services, we described the direction and size of the impact on ANC attendance, facility-based births, and PNC attendance, with the results tabulated. For facilitators and barriers to mHealth use, we inductively conducted a thematic analysis to identify barriers and facilitators to mHealth use by CHWs [40]. After thematic analysis, we synthesized and reflected on the results using the sociotechnical system (STS) framework developed by Davis et al [41]. The STS framework is not specific to CHWs or mHealth; however, it provides a useful lens for understanding how various dimensions—such as (1) people, (2) working practices, (3) program goals, (4) culture, (5) infrastructure, and (6) technology—influence the implementation and use of new technologies. The first 4 cover the social dimension of mHealth, while the last 2 cover the technology dimension of mHealth [42]. Applying the STS framework allowed a comprehensive and up-to-date analysis of the barriers and facilitators of both the technical and social systems of mHealth, rather than focusing on technology alone as has been done in other digital health studies [43,44]. Further discussion on the dimensions of the STS is presented in the Results section, while Figure 1 outlines details of the 6 dimensions of the STS framework.

Second, we explored within- and between-study relationships to describe the variability in the results before integrating and synthesizing the findings.

Finally, we assessed the robustness of the synthesis by reflecting on the methodology and the results in the Discussion section.

## Results

### Summary of the Studies

**Figure 2:**
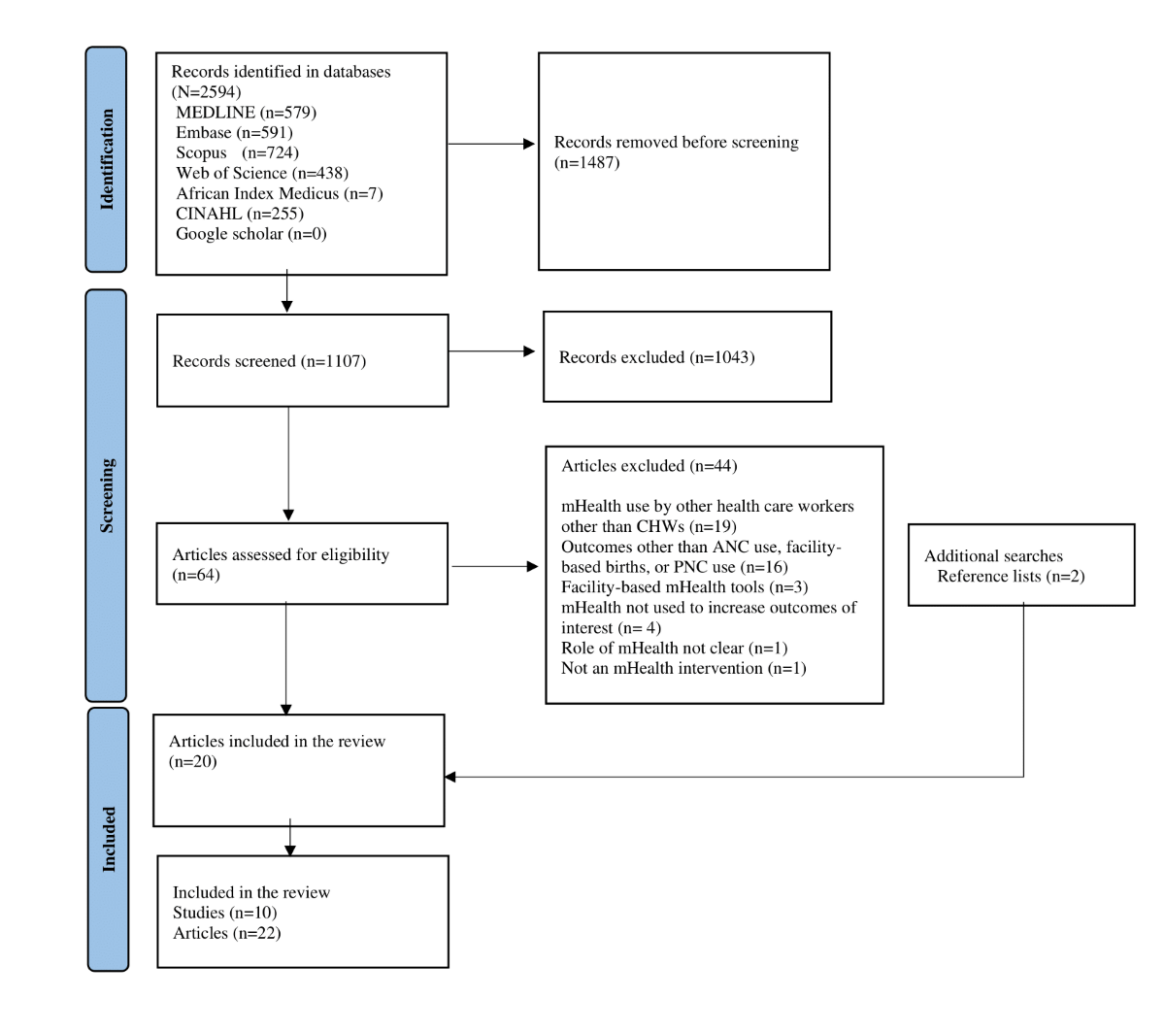
PRISMA (Preferred Reporting Items for Systematic Reviews and Meta-Analyses) flow diagram. ANC: antenatal care; CHW: community health worker; mHealth: mobile health; PNC: postnatal care.

We retrieved 2594 records from all databases (refer to Figure 2 for the PRISMA flow diagram). After removing 1487 (57.32%) duplicates from the 2594 records, 1107 (42.68%) articles underwent title and abstract screening. Of these 1107 articles, 1043 (94.22%) were excluded, leaving 64 (5.78%) articles. After full-text screening, 20 (31%) of the 64 articles were included. We found 2 additional articles from reference searching, resulting in a final number of 10 studies comprising 22 articles. The reasons for exclusion of 44 articles after full-text screening included mHealth use by health care workers other than CHWs (n=19, 43%); outcomes other than ANC use, facility-based births, or PNC use (n=16, 36%); facility-based mHealth tools (n=3, 7%); mHealth not used to improve the outcomes of interest (n=4, 9%); unclear role of mHealth (n=1, 2%); and not an mHealth intervention (n=1, 2%; Multimedia Appendix 3).

### Characteristics of the Studies

The 10 studies were published between 2012 and 2022 (refer to the Risk-of-Bias Assessment subsection for the study designs used). Of the 10 studies, 3 (30%) were published in Tanzania [45-47]; 2 (20%) each in Ethiopia [48,49] and Uganda [50,51]; and 1 (10%) each in Rwanda [52], Mozambique [53], and Kenya [54]. The mHealth program for Rwanda was implemented nationally, while the other mHealth programs were implemented either as a pilot or at a subnational level. Half of the mHealth platforms used were mobile apps (5/10, 50%) [45-47,49,53], followed by SMS text messaging–based platforms (4/10, 40%) [48,50,52,54] and voice calls (1/10, 10%) [51].

### Risk-of-Bias Assessment

All 22 included articles were assessed for risk of bias using the Mixed Methods Assessment Tool (Multimedia Appendix 4 [55-58]). Of the 4 qualitative articles, 3 (75%) were assessed as low risk of bias [55,56,59], and 1 (25%) had an unclear risk of bias [57]. Of the 4 RCTs, 1 (25%) had an unclear risk of bias [53], while 3 (75%) had a high risk of bias [45,48,51]. Of the 7 quantitative nonrandomized articles, 3 (43%) had a low risk of bias [46,52,60], while 4 (57%) had a high risk of bias [50,58,61,62]. Of the 2 descriptive articles, 1 (50%) had a low risk of bias [47], and 1 (50%) had a high risk of bias [63]. Finally, of the 5 mixed methods articles, 1 (20%) had a low risk of bias [54], while 4 (80%) had a high risk of bias [49,64-66].

### Impact of mHealth Use by CHWs on the Use of Maternal Health Services

#### Overview

The 10 included studies (13/22, 59% articles) reported at least 1 outcome of interest (Table 1). We present the findings based on the outcome of interest.

**Table 1:** Quantitative studies reporting on the impact of mobile health (mHealth) use by community health workers (CHWs).

Study, year; outcomes of interest	Study design	Country (context)	Intervention description	mHealth description	Main findings
Sevene et al [53], 2020; ANC^a^ and facility-based births	Cluster randomized controlled trial	Mozambique (subnational, rural, and urban districts)	Intervention: CHWs made home visits to pregnant women to provide education, danger signs identification, referrals, birth preparedness, and PNCb; CHWs also measured blood pressure and administered drugs for severe hypertensive disease; other interventions included transport support and health talks at health facilities Control: there were 6 control clusters where CHWs conducted home visits without using mHealth; no other interventions were provided	Type: Mobile app (PIERS^c^ On the Move) Use: the app was used for clinical decision support during the CHW home visit; CHWs used the app to identify pregnant women with danger signs (using pictograms), and if danger signs were present, they would refer the client to health facilities; if no danger signs were present, CHWs measured blood pressure, and if women met predefined criteria for referral, they would be referred immediately to an appropriate health facility	ANC: no difference. In comparison to control clusters, no difference in ≥4 ANC visits (48.6% vs 42.5%, aOR^d^ 1.57, 99% CI 0.97-2.52) Facility-based births: no difference. No differences in facility-based births (67.3% vs 74.2%, aOR 0.80, 99% CI 0.28-2.61; *P*=.71) or births at comprehensive emergency obstetric and neonatal care facilities (11.3% vs 13%, aOR 0.85, 99% CI 0.27-2.62; *P*=.70)
Hackett et al [45], 2018; facility-based births	Cluster randomized controlled trial	Tanzania (pilot and rural district)	Intervention: in 16 clusters, CHWs were trained in integrated maternal, neonatal, and child health; CHWs also conducted household visits to educate and refer clients to care during pregnancy and the postpartum period; mHealth app was used during the home visits Control: CHWs trained in integrated maternal, neonatal, and child health but used paper-based tools in 16 control clusters	Type: mobile app (developed using CommCare, an open-source platform designed specifically for use by frontline health workers) Use: CHWs used the app to register pregnant women, counsel pregnant women, identify danger signs, flag women who needed referrals, and create reminders for CHWs to follow up with the women they referred	Facility-based births: increase in use. A majority of pregnant women (74%) gave birth in transit or at the facility in the intervention villages compared with those in the control villages (62%); the odds of facility delivery were 2 times the odds between the intervention and control group (OR^e^ 1.96, CI 1.21-3.19; *P*=.01)
Atnafu et al [48], 2017; ANC and facility-based births	Cluster randomized controlled trial	Ethiopia (subnational, rural, and urban districts)	Intervention: the intervention targeted pregnant women in 2 clusters. Partial: higher cadre CHWs (ie, HEWs^f^) in 1 cluster were provided mHealth, while lower cadre volunteer CHWs were responsible for community mobilization, education, and referrals but were not provided mobile phones. Full: in 1 cluster, HEWs were provided mHealth, and volunteer CHWs were provided regular mobile phones for voice calls Control: no mHealth use by CHWs; CHWs performed home visits and community mobilization	Type: SMS text messaging based (FrontlineSMS) and regular mobile phone for voice calls Use: mHealth was used for SMS text messaging reminders to CHWs to follow up with pregnant women at 14, 24, 30, and 36 weeks of pregnancy to encourage them to attend all ANC visits and give birth at health facilities; in addition, voice calls were used to arrange referrals and communication between HEWs and volunteer CHWs; other functions included data collection and reporting as well as supply chain management	ANC: increase in the use of ANC. High ANC attendance at baseline at both intervention and control sites; significant increase in ≥4 ANC visits at intervention sites in comparison with control sites (partial intervention: 45.3%-59.8%; *P*<.001; full intervention: 15.8%-31.5%; *P*<.001; control sites: 24.5%-23.3%) Facility-based births: decrease in home births. Home births decreased at all intervention sites (partial intervention: 61.6%-33.7%; full intervention: 50.7%-35.8%; control sites: 72.8%-58.5%)
Ayiasi et al [51], 2016; ANC and facility-based births	Cluster randomized controlled trial	Uganda (pilot, 2 rural districts)	Intervention: existing CHWs (called village health teams) conducted 2 ANC home visits and 1 PNC home visit to provide standardized educational messages for maternal and newborn care in 8 clusters; in addition, each village health team had unlimited calls with health care workers for clinical consultation Control: village health teams in 8 health clusters provided usual community mobilization	Type: voice calls Use: mobile phones were used for clinical consultation between CHWs and providers and to arrange for referrals from the communities to health facilities	ANC: no significant differences in the rates of ANC visits. A majority of the women (85%) in the intervention clusters made ≥3 ANC visits compared with those in the control clusters (71%); aOR 1.82, 95% CI 0.65-5.09; *P*=.26) Facility-based births: higher at intervention sites than at control sites. Three times higher (90%) at the intervention sites than at the control sites (28%); the intervention increased the odds of facility-based births by 18-fold (OR 17.94, 95% CI 6.3-51.4; *P*<.001)
Webber et al [46], 2022; Webber et al [64], 2019; ANC, facility-based births, and PNC	Interrupted time series	Tanzania (subnational, rural)	Intervention: CHWs were trained to educate pregnant women on the importance of ANC, giving birth at facilities, and identifying danger signs using an mHealth app; other interventions implemented included birth kit distribution at 34 to 36 weeks’ gestation and transport support Control: CHWs who were not using mHealth	Type: mobile app (based on community health toolkit) Use: the mobile health app supported CHWs in providing education on the importance of attending ANC visits, giving birth at facilities, and identifying danger signs	Early results for facility-based births: suggested an increase in facility-based births. Rates of facility-based births increased (Bunda town from 87% to 93%, Bunda rural from 70% to 84%, and Tarime from 48% to 67%) between 2015 and 2016 Final evaluation for ANC: no difference. No difference in ≥4 ANC visits after introducing mHealth (immediate change: OR 1.19, 95% CI 0.93-1.51; *P*=.17; gradual effect: OR 1.02, 95% CI 0.99-1.05; *P*=.24) Final evaluation for facility-based births: increase in use. There was an increase in facility-based births from 71.8% at baseline to 85.1% after the intervention, with an immediate increase in the odds of facility-based births (OR 1.51, 95% CI 1.14-2.01; *P*=.004) and a small gradual effect (OR 1.03, 95% CI 1.00-1.07; *P*=.06). Final evaluation for PNC: no difference. No significant immediate change and a decline in the trend of PNC use after introducing mHealth (immediate change: OR 1.07, 95% CI 0.61-1.89; *P*=.81; gradual effect: OR 0.92, 95% CI 0.86-0.98; *P*=.01)
Hategeka et al [52], 2019; ANC, facility-based births, and PNC	Interrupted time series	Rwanda (national program, rural and urban districts)	Intervention: CHWs with mHealth supported pregnant women with education, follow-up, and linkage to care across the maternal health continuum. The intervention was stratified: Facilities in 20 districts received the usual support. Facilities in 10 districts received health system strengthening by 2 NGOs^g^ (including ongoing training for CHWs and equipment provision to health facilities) Control: CHWs not using mHealth	Type: SMS text messaging based Use: the open-source RapidSMS platform was used to facilitate communication between the health system and CHWs, facilitate clinical appointments by using reminders, support clinical decisions by providing information on what CHWs should do during an emergency, and facilitate referrals in emergencies by linking CHWs with the ambulance drivers; other functions of the RapidSMS system included registries or vital events tracking, data collection and reporting, and use an electronic health record system	Rwanda Demographic and Health Survey (2014-2015) data in 29 of the 30 districts: For ANC, no difference. No change in any ANC visits (immediate change: −1.00, 95% CI −2.30 to 0.29 and gradual effect: −0.04, 95% CI −0.14 to 0.06). No change in ≥4 ANC visits (immediate change: −1.69, 95% CI −9.94 to 6.55 and gradual effect: −0.40, 95% CI −1.09 to 0.27). No change in first trimester ANC visits (immediate change: −3.80, 95% CI −13.66 to 6.05 and gradual effect: −0.62, 95% CI −1.43 to 0.19). For facility-based births, no difference. No change in facility-based births; (immediate change: −1.79, 95% CI −6.16 to 2.58 and gradual effect: −0.13, 95% CI −0.49 to 0.22) Routine health facility data in all 30 districts: For ANC, no difference. No change in all 30 districts (*P*=.51 and *P*=.70 for supported districts and *P*=.38 and *P*=.50 for nonsupported districts). Facility-based births: a gradual increase in facility-based births. At the supported sites, there was no immediate change in facility-based births, but there was a change in gradual effect (estimate: 0.015 facility-based births per 1000 population, 95% CI 0.007-0.023; *P*<.001); no change in nonsupported sites. For PNC, an immediate increase in PNC visits. Change in supported facilities (immediate increase of 0.11 PNC visits per 1000 population, 95% CI 0.033-0.179; *P*=.007) and no change in trend; no change in the rate of PNC visits in nonsupported districts (*P*=.13)
Asiki et al [50], 2018; facility-based births	Nested cohort study	Uganda (pilot, rural)	Intervention: in 13 clusters, CHWs registered pregnancies and made monthly visits to pregnant women to relay SMS text messages and track outcomes Control: in 13 villages, CHWs followed pregnancies monthly using paper-based forms	Type: SMS text messaging based Use: mHealth supported CHWs to register pregnancies. In addition, each month, SMS text messaging reminders were sent to CHWs regarding which pregnant women they should visit to relay targeted messages on timely and safe ANC and facility-based births	Facility-based births: decreases in-home births. Intervention home delivery was 9.2%, and control home delivery was 22.4%; after controlling for confounders, the intervention arm had lower odds of home births (aOR 0.38, 95% CI 0.15-0.97)
Mushamiri et al [54], 2015; ANC	Mixed methods; the quantitative component used a nonrandomized control group study design	Kenya (subnational, rural)	Intervention: CHWs provided a community-based package of care and followed up with pregnant women using mHealth Control: CHWs provided a community-based package of care and did not use mHealth	Type: SMS text messaging–based platform Use: SMS text messaging reminders were sent to CHWs to remind pregnant women 3 days before the ANC visit once they were registered in the mHealth program; reminders were sent twice if the women failed to attend the ANC visit; reminders were sent up to 18 months after giving birth	ANC: mHealth increased the use of ANC. In comparison to the women not followed by CHWs using mHealth, the women in the intervention group had 3 times the odds of making more ANC visits, even after adjusting for HIV infection (aOR 2.58, 95% CI 1.10-6.01)
Nigussie et al [49], 2021; ANC, facility-based births, and PNC	Mixed methods	Ethiopia (rural and urban, subnational)	Intervention: CHWs registered, referred, followed up, and tracked pregnant women along the maternal health continuum of care; CHWs and their supervisors used mHealth to support their tasks	Type: mobile app Use: once clients were registered in the app, the system sent notifications and reminders to CHWs via the app to visit pregnant women and remind them to visit a health facility for ANC and PNC; these reminders were also sent through SMS text messaging to CHWs and clients; in addition, the program supported with free caller user group, facilitating referrals from the community to the facility	ANC: suggests increase in use. Pregnancy registration in the first and second trimesters increased between the third quarter of 2017 and the second quarter of 2018 (eg, first trimester registration increased from approximately 0% to approximately 10%), with a corresponding decline in registration in the third and fourth trimesters Facility-based births: suggests increase in use. Facility-based births increased from approximately 100 per quarter to >900 in the second quarter of 2018 PNC: suggests increase in use. PNC visits increased from approximately 100 per quarter to >700 in the second quarter of 2018
Fulcher et al [47], 2021 [65]; facility-based births, and PNC	Descriptive process evaluation	Tanzania (rural and urban, subnational)	Intervention: CHWs registered and enrolled pregnant women, as well as conducted 3 ANC home visits and 3 PNC visits using mHealth; other supporting interventions included community savings, transport support, and stakeholder engagement	Type: Mobile app (Mangologic app) Use: the app helped CHWs to know when to conduct home visits, identify pregnant women with danger signs and refer them to care, follow up with the women within 3 days of referral, and coordinate referrals with health facilities; the app was also used for data collection	Early findings for facility-based births: suggests increase in use. Among 13,231 births, 75% gave birth at hospitals in comparison with a baseline of 35%. Early findings for PNC: suggests increase in use. PNC attendance at intervention sites was 88% in comparison with a baseline of 36% from the demographic health survey Final evaluation for facility-based births: suggests increase in use. Health facility births increased from 60%, 70%, 80%, and 80% between years 1 and 4. Final evaluation for PNC: suggests increase in use. The number of women who attended PNC visits increased 60%, 60%, 70%, and 80% in years 1, 2, 3, and 4, respectively

^a^ANC: antenatal care.

^b^PNC: postnatal care.

^c^PIERS: preeclampsia integrated estimate of risk.

^d^aOR: adjusted odds ratio.

^e^OR: odds ratio.

^f^HEW: health extension worker.

^g^NGO: nongovernmental organization.

#### ANC Outcomes

Of the 10 studies, 7 (70%; 8/22, 36% articles) [46,48,49,51-54,60] reported at least 1 ANC outcome. On the basis of outcomes, only 2 (29%) of the 7 studies reported on ANC visits in the first trimester [49,52]. None of the studies reported on ≥8 ANC contacts. Of the 7 studies, 3 (43%) [48,49,54] showed an association between mHealth use by CHWs and increased ANC use. On the basis of platforms used, the studies that showed an association used SMS text messaging–based platforms (2/7, 29%) [48,54] and a mobile app (1/7, 14%) [49].

The observational study conducted in Ethiopia by Nigussie et al [49], which had a high risk of bias, suggested increased ANC use after mHealth use by CHWs. Using a mobile app, the study showed an increase in ANC contacts in the first trimester by approximately 10%.

A total of 3 RCTs (n=1, 33% with an unclear risk of bias [53]; n=2, 67% with a high risk of bias [48,51]) reported on ANC outcomes. Atnafu et al [48] used an SMS text messaging–based platform to remind CHWs to follow up pregnant women at 14, 24, 30, and 36 weeks of pregnancy in Ethiopia. The study found a significant increase in the proportion of women with ≥4 ANC visits at the intervention sites in comparison with the control sites (cluster with higher cadre CHWs using an SMS text messaging–based platform vs lower cadre CHWs who were not provided mobile phones: 45.3% to 59.8%; *P*<.001; cluster with higher cadre CHWs using an SMS text messaging–based platform vs lower cadre CHWs using mHealth for voice calls: 15.8% to 31.5%, *P*<.001; control sites [no mHealth]: 24.5% to 23.3%). The RCT conducted by Sevene et al [53] in Mozambique involved a clinical decision support mHealth app primarily used to support CHWs to screen for hypertension and make referrals to health facilities for care when they enrolled pregnant women during initial home visits or scheduled community follow-ups of pregnant women. As a secondary outcome, the proportion of women with ≥4 ANC visits was not statistically different between the intervention and control sites. Finally, the study conducted by Ayiasi et al [51] in Uganda, where CHWs used voice calls for consultations with health care workers during home visits, showed no significant difference in the number of women who attended ≥3 ANC visits between the intervention and control sites.

Three quasi-experimental studies (four articles) reported on ANC outcomes. Using an SMS text messaging–based mHealth platform in Kenya, Mushamiri et al [54] reported positive findings when comparing pregnant women receiving appointment reminders from CHWs using mHealth and pregnant women receiving CHW care without mHealth. In the study, women receiving care from CHWs using mHealth and starting ANC in the second trimester had 3 times the odds of attending ANC visits (adjusted odds ratio [OR] 2.58, 95% CI 1.10-6.01) than women receiving care from CHWs not using mHealth [54]. An SMS text messaging–based study (reported by 2 articles, 50% of the four articles that used quasi-experimental study designs) [52,60] used an interrupted time series design to evaluate a nationally implemented RapidSMS platform in Rwanda that enabled 2-way communication between CHWs and health care workers, facilitating clinical appointments of pregnant women by using reminders, supporting the clinical decisions by providing information on what CHWs should do during an emergency, and facilitating referrals during emergencies. After scaling the RapidSMS platform countrywide, 10 districts received health system–strengthening support from 2 nongovernmental organizations (NGOs; ongoing training provided to CHWs and equipment provided to health facilities). By contrast, the rest of the districts received the usual support from the Rwanda MOH. Using Rwanda Demographic and Health Survey (2014-2015) data in 29 of the 30 districts of Rwanda, Hategeka et al [52] found no change in any ANC visits, ANC visits in the first trimester, or ≥4 ANC visits. Using routinely collected health facility data in 461 health facilities, Ruton et al [60] found no change in ANC visits in all 30 districts of Rwanda. Finally, a quasi-experimental study with a time series design by Webber et al [46] used an mHealth app in Tanzania to educate women on the importance of attending maternal health services. The findings showed no significant differences in ≥4 ANC visits after introducing mHealth.

#### Facility-Based Births

Of the 10 studies, 9 (90%; 12/22, 55% articles) reported on facility-based births. Of these 9 studies, 8 (89%) found an association between mHealth use by CHWs and an increase in facility-based births or a reduction in home births. Of these 8 studies, 3 (38%) [48,49,54] used an SMS text messaging–based platform, 4 (50%) [45-47,49] used a mobile app, and 1 (12%) used voice calls [51].

A process evaluation study with a low risk of bias implemented in Tanzania by Fulcher et al [47], in which CHWs used a mobile app to increase demand for facility services by pregnant women, showed an increase in facility-based births from year 1 to year 4 of implementation (from 60% to 90%). Nigussie et al [49] also found an increase in facility-based births after the implementation of mHealth by CHWs.

In an RCT with a high risk of bias conducted in Tanzania, Hackett et al [45] compared the impact of the mHealth app in intervention clusters where CHWs were using the app and control clusters where CHWs were not using mHealth. The odds of facility-based births in the intervention clusters were double those in the control clusters (OR 1.96, 95% CI 1.21-3.19). The RCTs conducted by Atnafu et al [48] and Ayiasi et al [51] also reported a reduction in home births (home births decreased at all intervention sites [cluster with higher cadre CHWs using an SMS text messaging–based platform vs lower cadre CHWs not provided with mHealth: 61.6% to 33.7%; cluster with higher cadre CHWs using an SMS text messaging–based platform vs lower cadre CHWs using mHealth for voice calls: 50.7% to 35.8%; control sites: 72.8% to 58.5%]) and an increase in facility-based births (the intervention increased the odds of facility-based births by 18-fold [OR 17.94, 95% CI 6.3-51.4; *P*<.001]), respectively. However, Sevene et al [53] found no change in facility-based births between the intervention and control clusters.

Two quasi-experimental studies in which CHWs used mobile apps found a positive impact of mHealth on improving facility-based birth rates and reducing home birth rates. The study conducted in Tanzania by Webber et al [46], which had a low risk of bias, found that mHealth increased the odds of facility births (immediate increase: OR 1.51, 95% CI 1.14-2.01; *P*=.004; gradual effect: OR 1.03, 95% CI 1.00-1.07; *P*=.06). Asiki et al [50], who conducted a study in Uganda that had a high risk of bias, compared the impact of an SMS text messaging–based platform on CHWs using SMS text messaging reminders to follow up on pregnancy outcomes and CHWs not using mHealth. After controlling for confounders, mHealth reduced the odds of home births (adjusted OR 0.38, 95% CI 0.15-0.97). Two quasi-experimental articles based on the RapidSMS study in Rwanda showed mixed results. Hategeka et al [52] found no change in facility-based births. Ruton et al [60] found no change in the 20 districts that were not supported by the 2 NGOs, while there was a change in the 10 supported districts (gradual effect at the 10 supported sites: 0.015 facility-based births per 1000 population per month, 95% CI 0.007-0.023; *P*<.001), signifying the extra role played by embedding mHealth into broader health system–strengthening initiatives.

#### PNC Outcomes

Of the 10 studies, 2 (20%) observational studies [47,49] and 2 (20%) quasi-experimental studies reported on the impact of mHealth use by CHWs on PNC visits [46,60]. Of these 4 studies, 3 (75%) showed a positive association (n=2, 67% used a mobile app [47,49], and n=1, 33% used SMS text messaging [60]).

Fulcher et al [47] found that the mHealth app used by CHWs increased any PNC visits from 60% to 80% within 4 years of implementing the program. Nigussie et al [49] also showed an increase in any PNC visits after mHealth use by CHWs. In Rwanda, Ruton et al [60] reported an increase of 100% in PNC visits within a year of starting mHealth in the 10 districts that received extra NGO support (immediate increase of 0.11 PNC visits per 1000 population, 95% CI 0.033-0.179). However, the rate of PNC visits remained the same in the 20 districts not receiving health system strengthening. Finally, the study conducted in Tanzania by Webber et al [46] showed no impact of the mHealth app on PNC visits.

### Facilitators and Barriers to mHealth Use

#### Overview

Of the 10 studies, 8 (80%; 14/22, 64% articles) reported on facilitators and barriers to mHealth uptake (Table 2; Figure 1). We will discuss facilitators and barriers simultaneously and in alignment with the 6 dimensions of the STS framework developed by Davis et al [41]: program goals, people, culture, working practices, infrastructure, and technology. The definitions of the dimensions are provided in each subsection that follows.

**Table 2:** Studies reporting on the barriers and facilitators to mobile health (mHealth) use by community health worker (CHWs).

Study, year	Article, year	Study design	mHealth description	Study aims and findings
Sevene et al [53], 2020	Boene et al [66], 2021	Mixed methods	Mobile app	Facilitators: training on mHealth, refresher training, mentorship and supervision, improved status as health care workers through smartphone use, trust of communities, and good working relationships with clients Barriers: poor battery life, difficulty in securely attaching accessories, lack of power to charge mobile phones, and poor network connectivity
Atnafu et al [48], 2017	Atnafu [61], 2015; Atnafu and Bisrat [62], 2015	Cross-sectional	SMS text messaging based	Facilitators: good working relations with supervisors, as well as availability of free mobile phone and free airtime Barriers: poor network connectivity, lack of power to charge mobile phones, an inadequate number of mobile phones or other equipment, loss or damage of mobile phone and other equipment, and inadequate airtime
Ayiasi et al [51], 2016	Ayiasi et al [55], 2015	Qualitative	Voice calls	Facilitators: smartphones improved their status as health care workers, trust of communities, good working relations with supervisors, and positive supervisor feedback Barriers: staff absences, lack of power to charge mobile phones, poor network connectivity, poor attitude of health care workers, and poor relationships with clients
Webber et al [46], 2022	Webber et al [64], 2019; Webber et al [57], 2020	Mixed methods; qualitative	Mobile app	Facilitators: free mobile phone, airtime, and solar chargers Barriers: poor network connectivity, lack of power to charge mobile phones, inadequate airtime, poor app navigation. and poor app workflows
Hategeka et al [52], 2019	Ngabo et al [63], 2012; Musabyimana et al [56], 2018; Mwendwa [59], 2016; Mwendwa [58], 2018	Descriptive, qualitative; qualitative; cross-sectional	SMS text messaging based	Facilitators: MOHa ownership, incentives, strong existing CHW program, additional health system–strengthening activities, appropriate stakeholder engagement, high education level of CHWs, training, positive feedback, positive program outcomes, trust of communities, good working relations with supervisors, and good network connectivity Barriers: high initial cost of development, illiteracy, poor network connectivity, lack of power to charge mobile phones, inadequate airtime, high workload due to both paper- and smartphone-based data entry, poor organization of training, app in a foreign language, inadequate supervision, an inadequate number of mobile phones or other equipment, poor relationships with clients, loss or damage of mobile phone and other equipment, and no stipend or salary for CHWs
Nigussie et al [49], 2021	Nigussie et al [49], 2021	Mixed methods	Mobile app	Facilitators: appropriate stakeholder engagement and MOH ownership, as well as additional health system–strengthening activities Barriers: high workload due to both paper- and smartphone-based data entry, mobile phone–sharing culture, a fear of losing mobile phones to theft, delay in reporting the challenges of mHealth, a lack of skills in monitoring service delivery, loss or damage of mobile phone and other equipment, poor network connectivity, inadequate airtime, delay in repairing or replacing equipment, and burden of carrying multiple mobile devices
Fulcher et al [47], 2021	Fulcher et al [47], 2021	Descriptive process evaluation	Mobile app	Barriers: poor network connectivity, high loss to follow-up of clients, high attrition of CHWs, and software crashes
Mushamiri et al [54], 2015	Mushamiri et al [54], 2015	Mixed methods	SMS text messaging based	Facilitator: free closed user group calls and good network connectivity Barriers: contract termination with a network provider and late health-seeking behavior

^a^MOH: Ministry of Health.

#### People

The STS identifies this dimension as encompassing the users and stakeholders of a system and their characteristics. The included articles explored facilitators and barriers affecting CHWs, who are the main stakeholders, as well as their surrounding environment (eg, communities and supervisors), and their characteristics (eg, attitudes, behavior, and skills).

An unintended benefit yet powerful facilitator of mHealth use was the effect of the mHealth devices on the status of CHWs. mHealth improved the social status of CHWs because they were perceived as being recognized by the formal health system [55,66]. Perceived higher social status improved community trust, another facilitator identified in multiple studies [55,59,66]. Other facilitators identified included higher education level of CHWs [58,59], strong existing CHW program [63], MOH ownership of CHW programs [49,63], and positive feedback from supervisors [55,58,59].

The social dynamics within and outside the CHW program also positively or negatively impacted mHealth use. Good working relationships and positive feedback between CHWs, their supervisors, health care workers, and communities facilitated mHealth use [55,59,62,66]. By contrast, poor relationships among CHWs and communities and health care workers were singled out as barriers to mHealth use [49,55,56]. Other barriers identified in the studies included a lack of skills in monitoring service delivery by CHW supervisors [49], high staff absences and turnover [47,55], CHW illiteracy [59], poor attitude by facility-based staff [55], and high loss to follow-up of clients [47].

#### Working Practices

Processes refer to how practices and procedures are organized to support the system’s uptake. The included articles described the influence of systems, practices, and procedures designed to support CHWs in effectively using mHealth. Additional existing health system–strengthening activities facilitated positive outcomes in some mHealth-enabled CHW programs [49]; for example, additional training, provision of extra equipment and supplies, and supervision in Rwanda’s RapidSMS program facilitated mHealth use by CHWs [60]. In addition, engaging multiple and appropriate stakeholders, including telecommunication companies, facilitated the use of mHealth [49,63]. In a study in Kenya, suspending a contract with a mobile communication provider for a few weeks was one of the most significant barriers to SMS text messaging–based mHealth rollout [54].

Several other processes were reported in the studies. Adequate training and refresher training on mHealth [58,66], strong mentorship and supervision of CHWs [66], and enhancing the education levels of CHWs facilitated mHealth use [59]. By contrast, a high CHW workload [49], poor reporting systems [49,59], a lack of training or poor training organization [59], and inadequate supervision [59] were reported as barriers to mHealth use. The provision of incentives or salaries to CHWs was also mentioned, with regular incentives or salaries as a facilitator [63] and a lack of incentives or salaries or low incentives or salaries as a barrier to mHealth use [56].

#### Program Goals

This dimension explores how program performance and outcomes influence the uptake of a system. Very few studies reported on the impact of program goals on mHealth uptake by CHWs, and only 1 facilitator and 1 barrier were identified. Positive program outcomes (eg, he role played by CHWs in reducing mortality rates) in the RapidSMS program in Rwanda reinforced the use of mHealth [56,58,59]. The study conducted in Kenya reported late presentation to ANC as a barrier [54]. This factor had a detrimental impact on the mHealth-driven goals of ANC visits within the scope of this study.

#### Culture

In the STS framework, this dimension examines the influence of users’ and stakeholders’ beliefs, norms, and values within a system. Among the included studies, there was a lack of literature addressing how culture affected the use of mHealth. None of the studies reported on cultural factors that might facilitate mHealth adoption. The studies conducted in Ethiopia [49,62] identified the sharing of an mHealth-enabled mobile phone with other family members, a common norm, as a barrier to mHealth use because the mobile phone was often unavailable to CHWs when needed. In the study by Atnafu and Bisrat [62], more than a third of the CHWs reported sharing the mobile phone with other family members. In addition, the studies based on the RapidSMS platform in Rwanda cited the use of a foreign language (ie, English) in the app as a cultural barrier [58,59].

#### Infrastructure

This dimension encompasses the assets of a system. Among the included studies, mHealth equipment and internet connectivity were frequently noted as influential factors in the uptake of mHealth. Free mobile phones [57,62] as well as free airtime or reimbursement of airtime costs [57,62] were facilitators of mHealth use. The provision of solar power for charging devices was also reported as a facilitator [57]. Finally, reliable internet connectivity was mentioned as a facilitator of mHealth use [54,63].

Most of the studies focused on barriers associated with mHealth devices and related equipment. The most commonly reported challenges included loss or damage of mobile phones and other equipment [49,62,63], delays in repairing or replacing equipment [49], the burden of carrying multiple mobile devices [49], a fear of losing mobile phones to theft [49], an inadequate number of mobile phones or other equipment [56,62], poor quality of mobile phones and battery life [66], and difficulty in securely attaching mobile phone accessories [66]. In many settings, especially rural settings, the lack of electricity was commonly reported as a barrier [55,57,59,61-64,66]. Finally, poor mobile network connectivity [47,49,55,57,59,61,62,64,66] and inadequate airtime [49,59,61,64] were also mentioned as barriers to mHealth use.

#### Technology

This dimension focuses on how mHealth software influences the uptake of mHealth use. Free closed user group calls facilitated the use of a voice-based mHealth platform; no other facilitators were identified [54]. However, several technology-related barriers were reported, including poor app workflows [57,64] and frequent app software crashes [47]. The study by Ngabo et al [63] reported the high cost of mHealth technology as a barrier.

## Discussion

### Principal Findings

This review is the first to synthesize evidence on the use of mHealth by CHWs to improve the use of maternal health services in SSA. While the results of most of the studies (8/9, 89%) supported that mHealth use by CHWs increase facility-based births, they are mixed for ANC and PNC use. For ANC and PNC, only approximately half (3/7, 43%) and three quarters of the studies (3/4, 75%) showed that mHealth use by CHWs increased the use of these services, respectively. On the basis of the intervention descriptions, mHealth use by CHWs may have increased use by creating demand for these services. As shown previously, the demand created by mHealth use is possible through multiple pathways. mHealth use by CHWs may have increased the knowledge of good maternal health practices, leading to behavior change toward health facility use for care [47]. This is particularly important for studies that use mHealth apps. In addition, primarily through SMS text messaging–based platforms, reminders may have encouraged pregnant women’s attendance at health services [67]. It is also possible that mHealth may have increased demand by increasing satisfaction and trust in CHWs and the health system and may also have improved adherence to the practices used by CHWs to increase demand for health services [68].

This review adds evidence on the impact of mHealth use on the use of maternal health services. Previous reviews have focused on the impact of mHealth use on maternal health outcomes but did not distinguish the primary users of mHealth. A previous systematic review conducted by Gayesa et al [26] in low- and lower–middle-income countries found that mHealth use increased the odds of facility-based births and PNC use [26]. Similarly, Wagnew et al [69] reported that SMS text messaging–based mHealth increased the use of ≥4 ANC visits as well as facility-based births in low- and middle-income countries. Rahman et al [70] and Sondaal et al [27] also report positive effects of mHealth use on ANC coverage and facility-based births in low- and middle-income countries. However, our study is unique because it presents the effect of mHealth use specifically by CHWs, a target group not explored in the other studies. In addition, this is the first review to focus on SSA specifically.

We found mixed results on the impact of mHealth use by CHWs on ANC visits. Of the 7 studies that reported on ANC attendance, 3 (43%) showed that mHealth use by CHWs may increase ANC use. While the SMS text messaging–based mHealth studies in Kenya and Ethiopia [48,54] and the mobile app study in Ethiopia [49] found increases in the overall number of ANC visits or ≥4 ANC visits, all other studies (4/7, 57%) found no effect. We also observed that many studies reported ≥4 ANC visits as an outcome. Reporting on ≥4 ANC visits may be attributed to the previous WHO recommendation, according to which 4 ANC visits were deemed adequate [71,72]. Only 2 (29%) of the 7 studies reported on ANC visits in the first trimester, and none of the studies reported on ≥8 ANC contacts as recommended by recent WHO guidelines [73]. As observed in many settings, especially in SSA, women start ANC attendance late [74,75], which may affect the number of ANC visits; therefore, we suggest designing mHealth programs to specifically focus on encouraging early ANC attendance (Textbox 1). Designing mHealth to support women to start ANC attendance earlier may have 2 advantages. First, mHealth may help identify pregnancies early through decision support and referrals to health facilities. Second, mHealth may support the provision of high-quality, community-based ANC by CHWs [76]. This approach would ensure that mHealth supports the recommended ANC contacts as well as the quality and outcomes of these community contacts.

**A summary of recommendations for mobile health (mHealth) implementation in sub-Saharan Africa (SSA).**

**Maternal health continuum of care outcomes**

- Antenatal care (ANC): design mHealth programs measuring the impact of mHealth on ANC attendance in the first trimester and ≥8 ANC contacts.
- Postnatal care: design more studies to measure the impact of mHealth on postnatal care.

**Coverage of mHealth**

- National scale-up of mHealth programs is required in settings where mHealth has been shown to work but is being implemented as a pilot or at the subnational level.
- Where national scale-up of mHealth is desired, consider adding health system–strengthening activities in addition to mHealth intervention.
- Scale up mHealth platforms that have been shown to work in other settings and countries in SSA.

**Choice of mHealth platform**

- Consider SMS text messaging–based platforms and mobile apps.

**Sociotechnical system dimension requiring further data**

- Design studies to measure the influence of culture and program goals on mHealth use.

We found very few studies (4/10, 40%) reporting on PNC outcomes compared with the studies reporting on ANC (7/10, 70%) and facility-based birth (9/10, 90%) outcomes. Of the 4 studies, 1 (25%) SMS text messaging–based study [60] and 2 (50%) mobile app studies [47,49] found increases in PNC visits after implementing mHealth programs. We suggest more studies designed to specifically show the effect of mHealth on PNC visits because providing care during the postnatal period reduces maternal and neonatal deaths as well as complications [7] (Textbox 1).

The findings from this review also have implications for the scale-up of mHealth programs and mHealth platforms of choice in SSA. Of the 10 included studies, only 1 (10%), which was conducted in Rwanda, was implemented nationally [52], while the rest (n=9, 90%) were implemented as pilots or at the subnational level. In addition, the majority of the studies (9/10, 90%) used either SMS text messaging or an mHealth app. As the results of this review show the benefits of mHealth use by CHWs on maternal health outcomes, we suggest a national scale-up in pilot or subnational programs; alternatively, new programs may consider the scale-up of mHealth from the beginning. As a choice of platform, this review has shown that SMS text messaging–based platforms or mHealth apps may be used as the platforms of choice (Textbox 1).

This review has also identified facilitators and barriers to mHealth use by CHWs across the 6 dimensions of the STS framework. Most of the studies reported facilitators and barriers with regard to people, working practices, infrastructure, and technology among the 6 dimensions. One of the common findings concerned the perceived improvement in CHWs’ status when they started using mHealth, as well as improved trust. This finding is echoed across other studies in the literature [77,78] and may be an essential reason for introducing mHealth in limited-resource settings. A recent review on the use of mHealth by CHWs, specifically smart devices, also found that mHealth improves CHW status [79]. As echoed by Perry et al [31], improving the status of CHWs and increasing their recognition by the formal health system is a crucial enabler for successful CHW programs, and mHealth may provide the pathway to achieve this. In addition, paying attention to the social environment of CHWs, including relationships, plays a vital role in the success of mHealth programs [80].

The findings from the processes, infrastructure, and technology dimensions reinforce the importance of strengthening CHW programs and health service infrastructure before the introduction of mHealth or as part of its implementation. Critical components such as MOH ownership and stakeholder engagement, as well as standardized and robust systems such as training and the provision of incentives or salaries to CHWs need to be considered to ensure the success of mHealth in many settings. Other studies in the literature have also emphasized the need to build systems and appropriate governance to address barriers related to mHealth equipment and evolving mHealth technologies [81,82]. Therefore, we recommend that the design and implementation of mHealth programs in SSA should include health system–strengthening activities to maximize the impact of mHealth tools (Textbox 1).

We need further studies across the culture and goals dimensions of the STS framework. First, very few of the included studies reported on the influence of culture on mHealth use by CHWs in SSA. Incorporating various aspects of culture, such as the local language, into mHealth improves its acceptability, usability, and effectiveness [83,84]. Unfortunately, even in the literature, there are limited studies reporting on the impact of culture on mHealth use [84,85], and the results were similar in this review [58,59,62]. More studies should be designed to explore the impact of culture on mHealth use. Second, further research is needed on the impact of clearly defined goals as enablers and barriers to mHealth (Textbox 1).

### Limitations

This review has some limitations that should be considered when interpreting the findings. First, it focused on mHealth interventions by CHWs for women of reproductive age using ANC, giving birth at health facilities, and using PNC within 42 days in SSA. While this limits generalizability to other populations, settings, and maternal health outcomes, it allowed for a targeted examination of mHealth impact on key services across the continuum of care in a region with high maternal mortality.

Second, the review included studies that implemented mHealth alongside other health system–strengthening activities, making it difficult to isolate the effect of mHealth alone. However, this reflects the real-world implementation of mHealth as a tool to enhance CHW service delivery within broader health systems, rather than as a stand-alone solution.

Third, the review may have missed some relevant studies by excluding gray literature. In addition, not assessing publication bias could mean that the included studies are skewed toward positive findings. However, a comprehensive search of 6 databases and reference checking was conducted, and all studies meeting inclusion criteria from these sources were included.

Finally, studies were excluded if they lacked sufficient information on the mHealth intervention, which could introduce selection bias. However, this was necessary to ensure that the review could meaningfully synthesize and interpret how mHealth was used to impact outcomes.

### Recommendations

This study has implications for program implementation, policy, and research. Although our recommendations focus on mHealth implementation in SSA, we hope some lessons can be applied to other settings. We present a summary of recommendations in Textbox 1.

### Conclusions

The study found evidence that mHealth use by CHWs results in an increase in facility-based births. Although the results are mixed, approximately half of the studies (3/7, 43%) that reported on ANC and 75% (3/4) of studies that reported PNC attendance showed that mHealth use by CHWs increased the use of these services. We found limited studies (2/7, 29%) measuring the impact of mHealth on increasing ANC visits in the first trimester and no study reported on ≥8 ANC visits. On the basis of the STS framework, most of the studies explored barriers and facilitators across the people, processes and procedures, building and infrastructure, and technology dimensions. More studies are needed on the culture and goals dimensions of the STS to better understand the impact and uptake of mHealth for improving maternal health outcomes.

## Appendix

Multimedia Appendix 1 PRISMA (Preferred Reporting Items for Systematic Reviews and Meta-Analyses) checklist.

Multimedia Appendix 2 Search strategy.

Multimedia Appendix 3 Excluded studies and reasons for exclusion.

Multimedia Appendix 4 Risk-of-bias assessments.

## Abbreviations

ANC: antenatal care
CHW: community health worker
mHealth: mobile health
MMR: maternal mortality ratio
MOH: ministry of health
NGO: nongovernmental organization
OR: odds ratio
PNC: postnatal care
PRISMA: Preferred Reporting Items for Systematic Reviews and Meta-Analyses
RCT: randomized controlled trial
SSA: sub-Saharan Africa
STS: sociotechnical system
WHO: World Health Organization
