# Supplementary material for: Evaluating the Adoption of mHealth Technologies by Community Health Workers to Improve the Use of Maternal Health Services in Sub-Saharan Africa: Systematic Review
Source: JMIR Mhealth Uhealth. 2024 Sep 24;12:e55819. doi: 10.2196/55819 (PMC11462100; doi:10.2196/55819)
Supplement: Multimedia Appendix 2 [file mhealth_v12i1e55819_app2.docx]

# MULTIMEDIA Appendix 2 Search strategY

| **Database and search date** | **Number of articles found** |
| --- | --- |
| **Medline** | 579 |
| **Embase** | 591 |
| **Scopus** | 724 |
| **Web of Science** | 438 |
| **African Index Medicus** | 7 |
| **CINAHL** | 255 |
| **Google Scholar** | 0 |
| **Total** | 2594 |

## OVID/Medline (579)

| **1** | exp Pregnant Women/ or exp Pregnancy/ or Midwifery/ or Nurse Midwives/ or exp Postpartum Period/ or Maternal Health Services/ or exp Maternal-Child Health Services/ or exp Perinatal Care/ or exp Prenatal Care/ or exp Parturition/ or Reproductive Health Services/ or Reproductive Health/ or exp Labor, Obstetric/ or Maternal Death/ or (pregnan* or obstetr* or midwive* or midwife* or mid-wive* or mid-wife* or antenatal or postnatal or postpartum or post-natal or post-partum or gravid* or partur* or birth* or neonatal* or neo-natal* or lactat* or puerper* or labor or labour or term-birth or prenatal* or perinatal* or child-birth* or childbirth* or newborn* or (obstetr* adj5 deliver*) or breastfe* or breast-fe* or bottle-fe* or reproductive-health* or (maternal adj5 (health* or care* or welfare* or healthcare* or service* or death* or mortal*)) or reproductive-health* or reproductive-service* or pre-natal* or prenatal* or antenatal* or safe-motherhood* or safe-mother-hood or breech*).ti,ab,kf. | **1916641** |
| --- | --- | --- |
| **2** | Community Health Workers/ or (accompagnateur* or accredited-social-health-activist* or asha* or animator* or auxiliary-nurse* or allied-health* or basic-health-worker* or barefoot* or bare-foot* or birth-attendant* or bridge-to-health-team* or care-group* or case-coordinator* or child-health-worker* or community-health-worker* or chw* or close-to-community-provider* or community-agent* or community-aide* or community-based-practitioner* or community-case-management* or community-coordinator* or community-drug-distributor* or community-health-assistant* or community-health-aide* or community-health-agent* or community-health-care-provider* or community-healthcare-provider* or community-health-extension-worker* or community-health-nurse* or community-health-representative* or community-health-surveyor* or community-health-volunteer* or community-healthcare-worker* or community-health-care-worker* or community-healthcare-provider* or community-health-care-provider* or community-health-promoter* or community-health-care-provider* or community-liaison* or community-nutrition-worker* or community-practitioner* or community-resource-person* or community-surveillance-volunteer* or community-volunteer* or community-worker* or care-group* or dame-health-worker* or door-to-door* or extension-service* or extension-officer* or extension-staff* or extension-worker* or family-planning-agent* or family-advocate* or family-support-worker* or family-welfare-assistant* or family-welfare-worker* or field-based* or grassroots* or grass-roots* or hard-to-reach* or health-activist* or health-aide* or health-agent* or health-care-agent* or healthcare-agent* or health-assistant* or health-auxiliar* or health-care-worker* or healthcare-worker* or health-coach* or health-counselor* or health-development-army* or health-distributor* or health-education* or health-extension* or health-nurse* or health-officer* or health-motivator* or health-outreach* or health-out-reach* or health-promoter* or health-promotor* or health-surveillance-assistant* or health-visitor* or health-worker* or health-volunteer* or home-based-care* or home-care* or home-health* or home-service* or home-visit* or iccm* or imci* or intake-specialist* or lady-health-worker* or lay-aide* or lay-attendant* or lay-consultant* or lay-counselor* or lay-health-advisor* or lay-health-worker* or lhw* or lay-visitor* or lay-worker* or lead-mother* or link-worker* or malaria-agent* or child-health-worker* or child-health-worker* or medical-assistant* or midwife* or mid-wife* or midwive* or mid-wive* or mobile-clinic-team* or mother-coordinator* or mother-leader* or navigator* or nutrition-agent* or nutrition-counselor* or outreach-advocate* or outreach-case-manager* or outreach-educator* or outreach-worker* or out-reach-advocate* or out-reach-case-manager* or out-reach-educator* or out-reach-worker* or parent-liaison* or peer-advisor* or peer-counselor* or peer-educator* or peer-health-advisor* or peer-leader* or peer-supporter* or promotora* or rural-health-auxiliar* or support-worker* or surveillance-volunteer* or traditional-birth-attendant* or village-health-volunteer* or village-health-worker* or vhw* or village-drug-kit-manager* or village-health* or village-health-helper* or voluntary-health-worker* or voluntary-worker* or volunteer*).ti,ab,kf. | **425530** |
| **3** | exp Cell Phones/ or exp Computers, Handheld/ or exp Internet/ or exp Medical Informatics/ or exp Mobile Applications/ or exp Multimedia/ or exp Nursing informatics/ or exp Patient Portals/ or exp Public Health Informatics/ or exp Telemedicine/ or exp Telenursing/ or exp User-Computer Interface/ or (android or app or apps or cell-phone* or cellular-phone* or desktop* or desk-top* or digital-health or digital-diagnostic-device* or distance-consult* or distance-counsel* or distant-consult* or e-diagnos* or e-coach* or econsult* or e-consult* or ediagnos* or ehealth* or e-health* or exergam* or facebook or face-book or feature-phone* or fixed-laptop* or game or games or gamification or gaming or global-positioning-system* or gps or health-app* or health-kiosk* or health-technolog* or interactive-voice-response* or internet* or ipad or ipads or iphone* or i-pad or i-pads or i-phone* or laptop* or lap-top* or mhapp* or mh-app* or mhealth* or m-health* or mobile-app* or mobile-health* or mobile-technolog* or mobile-device* or mobile-phone* or palm-top* or palmtop* or patient-portal* or pda or pdas or personal-digital-assistant* or personal-electronic-health-record* or personal-health-record* or phone-app* or portable-media-player* or radio-frequency-identification* or rfid* or remote-consult* or remote-counsel* or satellite-phone* or serious-gam* or smartphone* or smart-phone* or smart-phone* or sms or social-media* or tablets or tablet or tele-app* or tele-care or tele-consult* or tele-counsel* or tele-diagnos* or tele-health or tele-medic* or tele-monitor* or tele-nursing or telecare or teleconsult* or telecounsel* or telediagnos* or telehealth* or telemedic* or telemonitor* or telenursing or telephone-app* or text-messag* or wearable* or web-portal* or webportal* or whatsapp* or whats-app* or world-wide-web or worldwideweb or www or nintendo or twitter or instagram or x-box or xbox or smart-watch*).ti,ab,kf. | **931604** |
| **4** | exp "Africa South of the Sahara"/ or (angol* or benin* or botswan* or burkina-faso* or burundi* or cabo-verd* or cameroon* or cape-verd* or central-african-republic* or chad* or comoros* or congo* or cote-d-ivoire* or cote-diIvoire* or djibouti* or equatorial-guinea* or eritrea* or eswatini* or ethiopia* or gabon* or gambia* or ghana or ghanes* or guinea* or ivory-coast* or kenya* or lesotho* or liberia* or madagasca* or malawi* or mali or malines* or mauritania* or mauriti* or mozambiq* or namibia* or niger or nigeria* or rhodesia* or ruand* or rwand* or sao-tome-and-principe* or sao-tome-principe* or senegal* or seychell* or sierra-leone* or somalia* or south-africa* or rio-muni or subsaharan-africa* or sub-saharan-africa* or subsahara-africa* or sub-sahara-africa* or sudan* or swaziland* or swazi-land* or tanzan* or togo or togoles* or ugand* or zambia* or zimbabw* or central-africa* or east-africa* or eastern-africa* or southern-africa* or west-africa* or western-africa* or africa-south-of-the-sahara).ti,ab,kf,pl,in. | **632201** |
| **5** | 1 and 2 and 3 and 4 | **580** |
| **6** | 5 not (exp Animals/ not exp Humans/) | **580** |
| **7** | 6 not case reports/ | **579** |

## Embase (591)

| **No.** | **Query** | **Results** |
| --- | --- | --- |
| **#9** | #7 NOT #8 | **591** |
| **#8** | #7 AND ('conference abstract'/it OR 'conference paper'/it OR 'conference review'/it) | **237** |
| **#7** | #6 NOT 'case report'/exp | **828** |
| **#6** | #5 NOT ([animals]/lim NOT [humans]/lim) | **840** |
| **#5** | #1 AND #2 AND #3 AND #4 | **841** |
| **#4** | 'africa south of the sahara'/exp OR angol*:ti,ab,kw,ff,ca OR benin*:ti,ab,kw,ff,ca OR botswan*:ti,ab,kw,ff,ca OR 'burkina faso*':ti,ab,kw,ff,ca OR burundi*:ti,ab,kw,ff,ca OR 'cabo verd*':ti,ab,kw,ff,ca OR cameroon*:ti,ab,kw,ff,ca OR 'cape verd*':ti,ab,kw,ff,ca OR 'central african republic*':ti,ab,kw,ff,ca OR chad*:ti,ab,kw,ff,ca OR comoros*:ti,ab,kw,ff,ca OR congo*:ti,ab,kw,ff,ca OR 'cote d ivoire*':ti,ab,kw,ff,ca OR 'cote diivoire*':ti,ab,kw,ff,ca OR djibouti*:ti,ab,kw,ff,ca OR 'equatorial guinea*':ti,ab,kw,ff,ca OR eritrea*:ti,ab,kw,ff,ca OR eswatini*:ti,ab,kw,ff,ca OR ethiopia*:ti,ab,kw,ff,ca OR gabon*:ti,ab,kw,ff,ca OR gambia*:ti,ab,kw,ff,ca OR ghana:ti,ab,kw,ff,ca OR ghanes*:ti,ab,kw,ff,ca OR guinea*:ti,ab,kw,ff,ca OR 'ivory coast*':ti,ab,kw,ff,ca OR kenya*:ti,ab,kw,ff,ca OR lesotho*:ti,ab,kw,ff,ca OR liberia*:ti,ab,kw,ff,ca OR madagasca*:ti,ab,kw,ff,ca OR malawi*:ti,ab,kw,ff,ca OR mali:ti,ab,kw,ff,ca OR malines*:ti,ab,kw,ff,ca OR mauritania*:ti,ab,kw,ff,ca OR mauriti*:ti,ab,kw,ff,ca OR mozambiq*:ti,ab,kw,ff,ca OR namibia*:ti,ab,kw,ff,ca OR niger:ti,ab,kw,ff,ca OR nigeria*:ti,ab,kw,ff,ca OR rhodesia*:ti,ab,kw,ff,ca OR ruand*:ti,ab,kw,ff,ca OR rwand*:ti,ab,kw,ff,ca OR 'sao tome and principe*':ti,ab,kw,ff,ca OR 'sao tome principe*':ti,ab,kw,ff,ca OR senegal*:ti,ab,kw,ff,ca OR seychell*:ti,ab,kw,ff,ca OR 'sierra leone*':ti,ab,kw,ff,ca OR somalia*:ti,ab,kw,ff,ca OR 'south africa*':ti,ab,kw,ff,ca OR 'rio muni':ti,ab,kw,ff,ca OR 'subsaharan africa*':ti,ab,kw,ff,ca OR 'sub saharan africa*':ti,ab,kw,ff,ca OR 'subsahara africa*':ti,ab,kw,ff,ca OR 'sub sahara africa*':ti,ab,kw,ff,ca OR sudan*:ti,ab,kw,ff,ca OR swaziland*:ti,ab,kw,ff,ca OR 'swazi land*':ti,ab,kw,ff,ca OR tanzan*:ti,ab,kw,ff,ca OR togo:ti,ab,kw,ff,ca OR togoles*:ti,ab,kw,ff,ca OR ugand*:ti,ab,kw,ff,ca OR zambia*:ti,ab,kw,ff,ca OR zimbabw*:ti,ab,kw,ff,ca OR 'central africa*':ti,ab,kw,ff,ca OR 'east africa*':ti,ab,kw,ff,ca OR 'eastern africa*':ti,ab,kw,ff,ca OR 'southern africa*':ti,ab,kw,ff,ca OR 'west africa*':ti,ab,kw,ff,ca OR 'western africa*':ti,ab,kw,ff,ca OR 'africa south of the sahara':ti,ab,kw,ff,ca | **693650** |
| **#3** | 'mobile phone'/exp OR 'personal digital assistant'/exp OR 'internet'/exp OR 'mass communication'/exp OR 'medical informatics'/exp OR 'mobile application'/exp OR 'multimedia'/exp OR 'nursing informatics'/exp OR 'telehealth'/exp OR android:ti,ab,kw OR app:ti,ab,kw OR apps:ti,ab,kw OR 'cell phone*':ti,ab,kw OR 'cellular phone*':ti,ab,kw OR desktop*:ti,ab,kw OR 'desk top*':ti,ab,kw OR 'digital health':ti,ab,kw OR 'digital diagnostic device*':ti,ab,kw OR 'distance consult*':ti,ab,kw OR 'distance counsel*':ti,ab,kw OR 'distant consult*':ti,ab,kw OR 'e diagnos*':ti,ab,kw OR 'e coach*':ti,ab,kw OR econsult*:ti,ab,kw OR 'e consult*':ti,ab,kw OR ediagnos*:ti,ab,kw OR ehealth*:ti,ab,kw OR 'e health*':ti,ab,kw OR exergam*:ti,ab,kw OR facebook:ti,ab,kw OR 'face book':ti,ab,kw OR 'feature phone*':ti,ab,kw OR game:ti,ab,kw OR games:ti,ab,kw OR gamification:ti,ab,kw OR gaming:ti,ab,kw OR 'global positioning system*':ti,ab,kw OR gps:ti,ab,kw OR 'health app*':ti,ab,kw OR 'health kiosk*':ti,ab,kw OR 'health technolog*':ti,ab,kw OR 'interactive voice response*':ti,ab,kw OR internet*:ti,ab,kw OR ipad:ti,ab,kw OR ipads:ti,ab,kw OR iphone*:ti,ab,kw OR 'i pad':ti,ab,kw OR 'i pads':ti,ab,kw OR 'i phone*':ti,ab,kw OR laptop*:ti,ab,kw OR 'lap top*':ti,ab,kw OR mhapp*:ti,ab,kw OR 'mh app*':ti,ab,kw OR mhealth*:ti,ab,kw OR 'm health*':ti,ab,kw OR 'mobile app*':ti,ab,kw OR 'mobile health*':ti,ab,kw OR 'mobile technolog*':ti,ab,kw OR 'mobile device*':ti,ab,kw OR 'mobile phone*':ti,ab,kw OR 'palm top*':ti,ab,kw OR palmtop*:ti,ab,kw OR 'patient portal*':ti,ab,kw OR pda:ti,ab,kw OR pdas:ti,ab,kw OR 'personal digital assistant*':ti,ab,kw OR 'personal electronic health record*':ti,ab,kw OR 'personal health record*':ti,ab,kw OR 'phone app*':ti,ab,kw OR 'portable media player*':ti,ab,kw OR 'radio frequency identification*':ti,ab,kw OR rfid*:ti,ab,kw OR 'remote consult*':ti,ab,kw OR 'remote counsel*':ti,ab,kw OR 'satellite phone*':ti,ab,kw OR 'serious gam*':ti,ab,kw OR smartphone*:ti,ab,kw OR 'smart phone*':ti,ab,kw OR sms:ti,ab,kw OR 'social media*':ti,ab,kw OR tablets:ti,ab,kw OR tablet:ti,ab,kw OR 'tele app*':ti,ab,kw OR 'tele care':ti,ab,kw OR 'tele consult*':ti,ab,kw OR 'tele counsel*':ti,ab,kw OR 'tele diagnos*':ti,ab,kw OR 'tele health':ti,ab,kw OR 'tele medic*':ti,ab,kw OR 'tele monitor*':ti,ab,kw OR 'tele nursing':ti,ab,kw OR telecare:ti,ab,kw OR teleconsult*:ti,ab,kw OR telecounsel*:ti,ab,kw OR telediagnos*:ti,ab,kw OR telehealth*:ti,ab,kw OR telemedic*:ti,ab,kw OR telemonitor*:ti,ab,kw OR telenursing:ti,ab,kw OR 'telephone app*':ti,ab,kw OR 'text messag*':ti,ab,kw OR wearable*:ti,ab,kw OR 'web portal*':ti,ab,kw OR webportal*:ti,ab,kw OR whatsapp*:ti,ab,kw OR 'whats app*':ti,ab,kw OR 'world wide web':ti,ab,kw OR worldwideweb:ti,ab,kw OR www:ti,ab,kw OR nintendo:ti,ab,kw OR twitter:ti,ab,kw OR instagram:ti,ab,kw OR 'x box':ti,ab,kw OR xbox:ti,ab,kw OR 'smart watch*':ti,ab,kw | **1097713** |
| **#2** | 'health auxiliary'/exp OR accompagnateur*:ti,ab,kw OR 'accredited social health activist*':ti,ab,kw OR asha*:ti,ab,kw OR animator*:ti,ab,kw OR 'auxiliary nurse*':ti,ab,kw OR 'allied health*':ti,ab,kw OR 'basic health worker*':ti,ab,kw OR barefoot*:ti,ab,kw OR 'bare foot*':ti,ab,kw OR 'birth attendant*':ti,ab,kw OR 'bridge to health team*':ti,ab,kw OR 'case coordinator*':ti,ab,kw OR 'community health worker*':ti,ab,kw OR chw*:ti,ab,kw OR 'close to community provider*':ti,ab,kw OR 'community agent*':ti,ab,kw OR 'community aide*':ti,ab,kw OR 'community based practitioner*':ti,ab,kw OR 'community case management*':ti,ab,kw OR 'community coordinator*':ti,ab,kw OR 'community drug distributor*':ti,ab,kw OR 'community health assistant*':ti,ab,kw OR 'community health aide*':ti,ab,kw OR 'community health agent*':ti,ab,kw OR 'community health extension worker*':ti,ab,kw OR 'community health nurse*':ti,ab,kw OR 'community health representative*':ti,ab,kw OR 'community health surveyor*':ti,ab,kw OR 'community health volunteer*':ti,ab,kw OR 'community healthcare worker*':ti,ab,kw OR 'community health care worker*':ti,ab,kw OR 'community healthcare provider*':ti,ab,kw OR 'community health promoter*':ti,ab,kw OR 'community health care provider*':ti,ab,kw OR 'community liaison*':ti,ab,kw OR 'community nutrition worker*':ti,ab,kw OR 'community practitioner*':ti,ab,kw OR 'community resource person*':ti,ab,kw OR 'community surveillance volunteer*':ti,ab,kw OR 'community volunteer*':ti,ab,kw OR 'community worker*':ti,ab,kw OR 'care group*':ti,ab,kw OR 'dame health worker*':ti,ab,kw OR 'door to door*':ti,ab,kw OR 'extension service*':ti,ab,kw OR 'extension officer*':ti,ab,kw OR 'extension staff*':ti,ab,kw OR 'extension worker*':ti,ab,kw OR 'family planning agent*':ti,ab,kw OR 'family advocate*':ti,ab,kw OR 'family support worker*':ti,ab,kw OR 'family welfare assistant*':ti,ab,kw OR 'family welfare worker*':ti,ab,kw OR 'field based*':ti,ab,kw OR grassroots*:ti,ab,kw OR 'grass roots*':ti,ab,kw OR 'hard to reach*':ti,ab,kw OR 'health activist*':ti,ab,kw OR 'health aide*':ti,ab,kw OR 'health agent*':ti,ab,kw OR 'health care agent*':ti,ab,kw OR 'healthcare agent*':ti,ab,kw OR 'health assistant*':ti,ab,kw OR 'health auxiliar*':ti,ab,kw OR 'health care worker*':ti,ab,kw OR 'healthcare worker*':ti,ab,kw OR 'health coach*':ti,ab,kw OR 'health counselor*':ti,ab,kw OR 'health development army*':ti,ab,kw OR 'health distributor*':ti,ab,kw OR 'health education*':ti,ab,kw OR 'health extension*':ti,ab,kw OR 'health nurse*':ti,ab,kw OR 'health officer*':ti,ab,kw OR 'health motivator*':ti,ab,kw OR 'health outreach*':ti,ab,kw OR 'health out reach*':ti,ab,kw OR 'health promoter*':ti,ab,kw OR 'health promotor*':ti,ab,kw OR 'health surveillance assistant*':ti,ab,kw OR 'health visitor*':ti,ab,kw OR 'health worker*':ti,ab,kw OR 'health volunteer*':ti,ab,kw OR 'home based care*':ti,ab,kw OR 'home care*':ti,ab,kw OR 'home health*':ti,ab,kw OR 'home service*':ti,ab,kw OR 'home visit*':ti,ab,kw OR iccm*:ti,ab,kw OR imci*:ti,ab,kw OR 'intake specialist*':ti,ab,kw OR 'lady health worker*':ti,ab,kw OR 'lay aide*':ti,ab,kw OR 'lay attendant*':ti,ab,kw OR 'lay consultant*':ti,ab,kw OR 'lay counselor*':ti,ab,kw OR 'lay health advisor*':ti,ab,kw OR 'lay health worker*':ti,ab,kw OR lhw*:ti,ab,kw OR 'lay visitor*':ti,ab,kw OR 'lay worker*':ti,ab,kw OR 'lead mother*':ti,ab,kw OR 'link worker*':ti,ab,kw OR 'malaria agent*':ti,ab,kw OR 'child health worker*':ti,ab,kw OR 'medical assistant*':ti,ab,kw OR midwife*:ti,ab,kw OR 'mid wife*':ti,ab,kw OR midwive*:ti,ab,kw OR 'mid wive*':ti,ab,kw OR 'mobile clinic team*':ti,ab,kw OR 'mother coordinator*':ti,ab,kw OR 'mother leader*':ti,ab,kw OR navigator*:ti,ab,kw OR 'nutrition agent*':ti,ab,kw OR 'nutrition counselor*':ti,ab,kw OR 'outreach advocate*':ti,ab,kw OR 'outreach case manager*':ti,ab,kw OR 'outreach educator*':ti,ab,kw OR 'outreach worker*':ti,ab,kw OR 'out reach advocate*':ti,ab,kw OR 'out reach case manager*':ti,ab,kw OR 'out reach educator*':ti,ab,kw OR 'out reach worker*':ti,ab,kw OR 'parent liaison*':ti,ab,kw OR 'peer advisor*':ti,ab,kw OR 'peer counselor*':ti,ab,kw OR 'peer educator*':ti,ab,kw OR 'peer health advisor*':ti,ab,kw OR 'peer leader*':ti,ab,kw OR 'peer supporter*':ti,ab,kw OR promotora*:ti,ab,kw OR 'rural health auxiliar*':ti,ab,kw OR 'support worker*':ti,ab,kw OR 'surveillance volunteer*':ti,ab,kw OR 'traditional birth attendant*':ti,ab,kw OR 'village health volunteer*':ti,ab,kw OR 'village health worker*':ti,ab,kw OR vhw*:ti,ab,kw OR 'village drug kit manager*':ti,ab,kw OR 'village health*':ti,ab,kw OR 'village health helper*':ti,ab,kw OR 'voluntary health worker*':ti,ab,kw OR 'voluntary worker*':ti,ab,kw OR volunteer*:ti,ab,kw | **547140** |
| **#1** | 'pregnant woman'/exp OR 'pregnancy'/exp OR 'midwife'/exp OR 'puerperium'/exp OR 'maternal health service'/exp OR 'maternal child health care'/exp OR 'maternal care'/de OR 'perinatal care'/de OR 'prenatal care'/de OR 'prenatal screening'/exp OR 'birth'/exp OR 'reproductive health'/exp OR 'childbirth'/exp OR 'maternal death'/exp OR pregnan*:ti,ab,kw OR obstetr*:ti,ab,kw OR midwive*:ti,ab,kw OR midwife*:ti,ab,kw OR 'mid wive*':ti,ab,kw OR 'mid wife*':ti,ab,kw OR antenatal:ti,ab,kw OR postnatal:ti,ab,kw OR postpartum:ti,ab,kw OR 'post natal':ti,ab,kw OR 'post partum':ti,ab,kw OR gravid*:ti,ab,kw OR partur*:ti,ab,kw OR birth*:ti,ab,kw OR neonatal*:ti,ab,kw OR 'neo natal*':ti,ab,kw OR lactat*:ti,ab,kw OR puerper*:ti,ab,kw OR labor:ti,ab,kw OR labour:ti,ab,kw OR 'term birth':ti,ab,kw OR perinatal*:ti,ab,kw OR 'child birth*':ti,ab,kw OR childbirth*:ti,ab,kw OR newborn*:ti,ab,kw OR ((obstetr* NEAR/5 deliver*):ti,ab,kw) OR breastfe*:ti,ab,kw OR 'breast fe*':ti,ab,kw OR 'bottle fe*':ti,ab,kw OR ((maternal NEAR/5 (health* OR care* OR welfare* OR healthcare* OR service* OR death* OR mortal*)):ti,ab,kw) OR 'reproductive health*':ti,ab,kw OR 'reproductive service*':ti,ab,kw OR 'pre natal*':ti,ab,kw OR prenatal*:ti,ab,kw OR antenatal*:ti,ab,kw OR 'safe motherhood*':ti,ab,kw OR 'safe mother hood':ti,ab,kw OR breech*:ti,ab,kw | **2319463** |

## Elsevier/Scopus (724)

| **History Count** | **Search Terms** | **Results** |
| --- | --- | --- |
| **#9** | #8 AND (LIMIT-TO (DOCTYPE , "ar") OR LIMIT-TO (DOCTYPE , "re") OR LIMIT-TO (DOCTYPE , "ed") OR LIMIT-TO (DOCTYPE , "er") OR LIMIT-TO (DOCTYPE , "sh") OR LIMIT-TO (DOCTYPE , "ch") OR LIMIT-TO (DOCTYPE , "le")) | **724** |
| **#8** | #2 AND #3 AND #4 AND #7 | **774** |
| **#7** | #5 OR #6 | **1,632,338** |
| **#6** | AFFILCOUNTRY ((angol* OR benin* OR botswan* OR burkina-faso* OR burundi* OR cabo-verd* OR cameroon* OR cape-verd* OR central-african-republic* OR chad* OR comoros* OR congo* OR cote-d-ivoire* OR cote-diivoire* OR djibouti* OR equatorial-guinea* OR eritrea* OR eswatini* OR ethiopia* OR gabon* OR gambia* OR ghana OR ghanes* OR guinea* OR ivory-coast* OR kenya* OR lesotho* OR liberia* OR madagasca* OR malawi* OR mali OR malines* OR mauritania* OR mauriti* OR mozambiq* OR namibia* OR niger OR nigeria* OR rhodesia* OR ruand* OR rwand* OR sao-tome-and-principe* OR sao-tome-principe* OR senegal* OR seychell* OR sierra-leone* OR somalia* OR south-africa* OR rio-muni OR subsaharan-africa* OR sub-saharan-africa* OR subsahara-africa* OR sub-sahara-africa* OR sudan* OR swaziland* OR swazi-land* OR tanzan* OR togo OR togoles* OR ugand* OR zambia* OR zimbabw* OR central-africa* OR east-africa* OR eastern-africa* OR southern-africa* OR west-africa* OR western-africa* OR africa-south-of-the-sahara)) | **984,555** |
| **#5** | TITLE-ABS-KEY ((angol* OR benin* OR botswan* OR burkina-faso* OR burundi* OR cabo-verd* OR cameroon* OR cape-verd* OR central-african-republic* OR chad* OR comoros* OR congo* OR cote-d-ivoire* OR cote-diivoire* OR djibouti* OR equatorial-guinea* OR eritrea* OR eswatini* OR ethiopia* OR gabon* OR gambia* OR ghana OR ghanes* OR guinea* OR ivory-coast* OR kenya* OR lesotho* OR liberia* OR madagasca* OR malawi* OR mali OR malines* OR mauritania* OR mauriti* OR mozambiq* OR namibia* OR niger OR nigeria* OR rhodesia* OR ruand* OR rwand* OR sao-tome-and-principe* OR sao-tome-principe* OR senegal* OR seychell* OR sierra-leone* OR somalia* OR south-africa* OR rio-muni OR subsaharan-africa* OR sub-saharan-africa* OR subsahara-africa* OR sub-sahara-africa* OR sudan* OR swaziland* OR swazi-land* OR tanzan* OR togo OR togoles* OR ugand* OR zambia* OR zimbabw* OR central-africa* OR east-africa* OR eastern-africa* OR southern-africa* OR west-africa* OR western-africa* OR africa-south-of-the-sahara)) | **1,129,555** |
| **#4** | TITLE-ABS-KEY ((android OR app OR apps OR cell-phone* OR cellular-phone* OR desktop* OR desk-top* OR digital-health OR digital-diagnostic-device* OR distance-consult* OR distance-counsel* OR distant-consult* OR e-diagnos* OR e-coach* OR econsult* OR e-consult* OR ediagnos* OR ehealth* OR e-health* OR exergam* OR facebook OR face-book OR feature-phone* OR game OR games OR gamification OR gaming OR global-positioning-system* OR gps OR health-app* OR health-kiosk* OR health-technolog* OR interactive-voice-response* OR internet* OR ipad OR ipads OR iphone* OR i-pad OR i-pads OR i-phone* OR laptop* OR lap-top* OR mhapp* OR mh-app* OR mhealth* OR m-health* OR mobile-app* OR mobile-health* OR mobile-technolog* OR mobile-device* OR mobile-phone* OR palm-top* OR palmtop* OR patient-portal* OR pda OR pdas OR personal-digital-assistant* OR personal-electronic-health-record* OR personal-health-record* OR phone-app* OR portable-media-player* OR radio-frequency-identification* OR rfid* OR remote-consult* OR remote-counsel* OR satellite-phone* OR serious-gam* OR smartphone* OR smart-phone* OR smart-phone* OR sms OR social-media* OR tablets OR tablet OR tele-app* OR tele-care OR tele-consult* OR tele-counsel* OR tele-diagnos* OR tele-health OR tele-medic* OR tele-monitor* OR tele-nursing OR telecare OR teleconsult* OR telecounsel* OR telediagnos* OR telehealth* OR telemedic* OR telemonitor* OR telenursing OR telephone-app* OR text-messag* OR wearable* OR web-portal* OR webportal* OR whatsapp* OR whats-app* OR world-wide-web OR worldwideweb OR www OR nintendo OR twitter OR instagram OR x-box OR xbox OR smart-watch*)) | **2,279,082** |
| **#3** | TITLE-ABS-KEY ((accompagnateur* OR accredited-social-health-activist* OR asha* OR animator* OR auxiliary-nurse* OR allied-health* OR basic-health-worker* OR barefoot* OR bare-foot* OR birth-attendant* OR bridge-to-health-team* OR care-group* OR case-coordinator* OR child-health-worker* OR community-health-worker* OR chw* OR close-to-community-provider* OR community-agent* OR community-aide* OR community-based-practitioner* OR community-case-management* OR community-coordinator* OR community-drug-distributor* OR community-health-assistant* OR community-health-aide* OR community-health-agent* OR community-health-care-provider* OR community-healthcare-provider* OR community-health-extension-worker* OR community-health-nurse* OR community-health-representative* OR community-health-surveyor* OR community-health-volunteer* OR community-healthcare-worker* OR community-health-care-worker* OR community-healthcare-provider* OR community-health-care-provider* OR community-health-promoter* OR community-health-care-provider* OR community-liaison* OR community-nutrition-worker* OR community-practitioner* OR community-resource-person* OR community-surveillance-volunteer* OR community-volunteer* OR community-worker* OR care-group* OR dame-health-worker* OR door-to-door* OR extension-service* OR extension-officer* OR extension-staff* OR extension-worker* OR family-planning-agent* OR family-advocate* OR family-support-worker* OR family-welfare-assistant* OR family-welfare-worker* OR field-based* OR grassroots* OR grass-roots* OR hard-to-reach* OR health-activist* OR health-aide* OR health-agent* OR health-care-agent* OR healthcare-agent* OR health-assistant* OR health-auxiliar* OR health-care-worker* OR healthcare-worker* OR health-coach* OR health-counselor* OR health-development-army* OR health-distributor* OR health-education* OR health-extension* OR health-nurse* OR health-officer* OR health-motivator* OR health-outreach* OR health-out-reach* OR health-promoter* OR health-promotor* OR health-surveillance-assistant* OR health-visitor* OR health-worker* OR health-volunteer* OR home-based-care* OR home-care* OR home-health* OR home-service* OR home-visit* OR iccm* OR imci* OR intake-specialist* OR lady-health-worker* OR lay-aide* OR lay-attendant* OR lay-consultant* OR lay-counselor* OR lay-health-advisor* OR lay-health-worker* OR lhw* OR lay-visitor* OR lay-worker* OR lead-mother* OR link-worker* OR malaria-agent* OR child-health-worker* OR child-health-worker* OR medical-assistant* OR midwife* OR mid-wife* OR midwive* OR mid-wive* OR mobile-clinic-team* OR mother-coordinator* OR mother-leader* OR navigator* OR nutrition-agent* OR nutrition-counselor* OR outreach-advocate* OR outreach-case-manager* OR outreach-educator* OR outreach-worker* OR out-reach-advocate* OR out-reach-case-manager* OR out-reach-educator* OR out-reach-worker* OR parent-liaison* OR peer-advisor* OR peer-counselor* OR peer-educator* OR peer-health-advisor* OR peer-leader* OR peer-supporter* OR promotora* OR rural-health-auxiliar* OR support-worker* OR surveillance-volunteer* OR traditional-birth-attendant* OR village-health-volunteer* OR village-health-worker* OR vhw* OR village-drug-kit-manager* OR village-health* OR village-health-helper* OR voluntary-health-worker* OR voluntary-worker* OR volunteer*)) | **820,082** |
| **#2** | TITLE-ABS-KEY ((pregnan* OR obstetr* OR midwive* OR midwife* OR mid-wive* OR mid-wife* OR antenatal OR postnatal OR postpartum OR post-natal OR post-partum OR gravid* OR partur* OR birth* OR neonatal* OR neo-natal* OR lactat* OR puerper* OR labor OR labour OR term-birth OR prenatal* OR perinatal* OR child-birth* OR childbirth* OR newborn* OR (obstetr* W/5 deliver*) OR breastfe* OR breast-fe* OR bottle-fe* OR reproductive-health* OR (maternal W/5 (health* OR care* OR welfare* OR healthcare* OR service* OR death* OR mortal*)) OR reproductive-health* OR reproductive-service* OR pre-natal* OR prenatal* OR antenatal* OR safe-motherhood* OR safe-mother-hood OR breech*)) | **3,202,624** |

## Clarivate Analytics/Web of Science core Collection (438)

| **5** | **#4 AND #3 AND #2 AND #1** | **438** |
| --- | --- | --- |
| **4** | **TS=("angol*" OR "benin*" OR "botswan*" OR "burkina faso*" OR "burundi*" OR "cabo verd*" OR "cameroon*" OR "cape verd*" OR "central african republic*" OR "chad*" OR "comoros*" OR "congo*" OR "cote d ivoire*" OR "cote diIvoire*" OR "djibouti*" OR "equatorial guinea*" OR "eritrea*" OR "eswatini*" OR "ethiopia*" OR "gabon*" OR "gambia*" OR "ghana" OR "ghanes*" OR "guinea*" OR "ivory coast*" OR "kenya*" OR "lesotho*" OR "liberia*" OR "madagasca*" OR "malawi*" OR "mali" OR "malines*" OR "mauritania*" OR "mauriti*" OR "mozambiq*" OR "namibia*" OR "niger" OR "nigeria*" OR "rhodesia*" OR "ruand*" OR "rwand*" OR "sao tome and principe*" OR "sao tome principe*" OR "senegal*" OR "seychell*" OR "sierra leone*" OR "somalia*" OR "south africa*" OR "rio muni" OR "subsaharan africa*" OR "sub saharan africa*" OR "subsahara africa*" OR "sub sahara africa*" OR "sudan*" OR "swaziland*" OR "swazi land*" OR "tanzan*" OR "togo" OR "togoles*" OR "ugand*" OR "zambia*" OR "zimbabw*" OR "central africa*" OR "east africa*" OR "eastern africa*" OR "southern africa*" OR "west africa*" OR "western africa*" OR "africa south of the sahara")** | **891,369** |
| **3** | **TS=("android" OR "app" OR "apps" OR "cell phone*" OR "cellular phone*" OR "desktop*" OR "desk top*" OR "digital health" OR "digital diagnostic device*" OR "distance consult*" OR "distance counsel*" OR "distant consult*" OR "e diagnos*" OR "e coach*" OR "econsult*" OR "e consult*" OR "ediagnos*" OR "ehealth*" OR "e health*" OR "exergam*" OR "facebook" OR "face book" OR "feature phone*" OR "game" OR "games" OR "gamification" OR "gaming" OR "global positioning system*" OR "gps" OR "health app*" OR "health kiosk*" OR "health technolog*" OR "interactive voice response*" OR "internet*" OR "ipad" OR "ipads" OR "iphone*" OR "i pad" OR "i pads" OR "i phone*" OR "laptop*" OR "lap top*" OR "mhapp*" OR "mh app*" OR "mhealth*" OR "m health*" OR "mobile app*" OR "mobile health*" OR "mobile technolog*" OR "mobile device*" OR "mobile phone*" OR "palm top*" OR "palmtop*" OR "patient portal*" OR "pda" OR "pdas" OR "personal digital assistant*" OR "personal electronic health record*" OR "personal health record*" OR "phone app*" OR "portable media player*" OR "radio frequency identification*" OR "rfid*" OR "remote consult*" OR "remote counsel*" OR "satellite phone*" OR "serious gam*" OR "smartphone*" OR "smart phone*" OR "smart phone*" OR "sms" OR "social media*" OR "tablets" OR "tablet" OR "tele app*" OR "tele care" OR "tele consult*" OR "tele counsel*" OR "tele diagnos*" OR "tele health" OR "tele medic*" OR "tele monitor*" OR "tele nursing" OR "telecare" OR "teleconsult*" OR "telecounsel*" OR "telediagnos*" OR "telehealth*" OR "telemedic*" OR "telemonitor*" OR "telenursing" OR "telephone app*" OR "text messag*" OR "wearable*" OR "web portal*" OR "webportal*" OR "whatsapp*" OR "whats app*" OR "world wide web" OR "worldwideweb" OR "www" OR "nintendo" OR "twitter" OR "instagram" OR "x box" OR "xbox" OR "smart watch*")** | **1,043,888** |
| **2** | **TS=("accompagnateur*" OR "accredited social health activist*" OR "asha*" OR "animator*" OR "auxiliary nurse*" OR "allied health*" OR "basic health worker*" OR "barefoot*" OR "bare foot*" OR "birth attendant*" OR "bridge to health team*" OR "care group*" OR "case coordinator*" OR "child health worker*" OR "community health worker*" OR "chw*" OR "close to community provider*" OR "community agent*" OR "community aide*" OR "community based practitioner*" OR "community case management*" OR "community coordinator*" OR "community drug distributor*" OR "community health assistant*" OR "community health aide*" OR "community health agent*" OR "community health care provider*" OR "community healthcare provider*" OR "community health extension worker*" OR "community health nurse*" OR "community health representative*" OR "community health surveyor*" OR "community health volunteer*" OR "community healthcare worker*" OR "community health care worker*" OR "community healthcare provider*" OR "community health care provider*" OR "community health promoter*" OR "community health care provider*" OR "community liaison*" OR "community nutrition worker*" OR "community practitioner*" OR "community resource person*" OR "community surveillance volunteer*" OR "community volunteer*" OR "community worker*" OR "care group*" OR "dame health worker*" OR "door to door*" OR "extension service*" OR "extension officer*" OR "extension staff*" OR "extension worker*" OR "family planning agent*" OR "family advocate*" OR "family support worker*" OR "family welfare assistant*" OR "family welfare worker*" OR "field based*" OR "grassroots*" OR "grass roots*" OR "hard to reach*" OR "health activist*" OR "health aide*" OR "health agent*" OR "health care agent*" OR "healthcare agent*" OR "health assistant*" OR "health auxiliar*" OR "health care worker*" OR "healthcare worker*" OR "health coach*" OR "health counselor*" OR "health development army*" OR "health distributor*" OR "health education*" OR "health extension*" OR "health nurse*" OR "health officer*" OR "health motivator*" OR "health outreach*" OR "health out reach*" OR "health promoter*" OR "health promotor*" OR "health surveillance assistant*" OR "health visitor*" OR "health worker*" OR "health volunteer*" OR "home based care*" OR "home care*" OR "home health*" OR "home service*" OR "home visit*" OR "iccm*" OR "imci*" OR "intake specialist*" OR "lady health worker*" OR "lay aide*" OR "lay attendant*" OR "lay consultant*" OR "lay counselor*" OR "lay health advisor*" OR "lay health worker*" OR "lhw*" OR "lay visitor*" OR "lay worker*" OR "lead mother*" OR "link worker*" OR "malaria agent*" OR "child health worker*" OR "child health worker*" OR "medical assistant*" OR "midwife*" OR "mid wife*" OR "midwive*" OR "mid wive*" OR "mobile clinic team*" OR "mother coordinator*" OR "mother leader*" OR "navigator*" OR "nutrition agent*" OR "nutrition counselor*" OR "outreach advocate*" OR "outreach case manager*" OR "outreach educator*" OR "outreach worker*" OR "out reach advocate*" OR "out reach case manager*" OR "out reach educator*" OR "out reach worker*" OR "parent liaison*" OR "peer advisor*" OR "peer counselor*" OR "peer educator*" OR "peer health advisor*" OR "peer leader*" OR "peer supporter*" OR "promotora*" OR "rural health auxiliar*" OR "support worker*" OR "surveillance volunteer*" OR "traditional birth attendant*" OR "village health volunteer*" OR "village health worker*" OR "vhw*" OR "village drug kit manager*" OR "village health*" OR "village health helper*" OR "voluntary health worker*" OR "voluntary worker*" OR "volunteer*")** | **451,628** |
| **1** | **TS=("pregnan*" OR "obstetr*" OR "midwive*" OR "midwife*" OR "mid wive*" OR "mid wife*" OR "antenatal" OR "postnatal" OR "postpartum" OR "post natal" OR "post partum" OR "gravid*" OR "partur*" OR "birth*" OR "neonatal*" OR "neo natal*" OR "lactat*" OR "puerper*" OR "labor" OR "labour" OR "term birth" OR "prenatal*" OR "perinatal*" OR "child birth*" OR "childbirth*" OR "newborn*" OR ("obstetr*" NEAR/5 "deliver*") OR "breastfe*" OR "breast fe*" OR "bottle fe*" OR "reproductive health*" OR ("maternal" NEAR/5 ("health*" OR "care*" OR "welfare*" OR "healthcare*" OR "service*" OR "death*" OR "mortal*")) OR "reproductive health*" OR "reproductive service*" OR "pre natal*" OR "prenatal*" OR "antenatal*" OR "safe motherhood*" OR "safe mother hood" OR "breech*")** | **1,945,004** |

## WHO/African Index Medicus (7)

1. **Maternal health (services)**

pregnan* OR obstetr* OR midwive* OR midwife* OR "mid wive" OR "mid wife" OR "mid wives" OR "mid wifes" OR antenatal OR postnatal* OR postpartum OR "post natal" OR "post partum" OR gravid* OR partur* OR birth* OR neonatal* OR "neo natal" OR lactat* OR puerper* OR labor OR labour OR "term birth" OR "term births" OR prenatal* OR perinatal* OR "child birth" OR childbirth* OR newborn* OR breastfe* OR "breast feeding" OR "bottle feeding" OR "reproductive health" OR "maternal health" OR "maternal care" OR "maternal welfare" OR "maternal healthcare" OR "maternal service" OR "maternal death" OR "maternal services" OR "maternal deaths" OR "maternal mortality" OR "reproductive service" OR "pre natal" OR prenatal* OR antenatal* OR "safe motherhood" OR "safe mother hood" OR breech*

1. **Community health workers (CHW)**

accompagnateur OR "accredited social health activist" OR asha OR animator* OR "auxiliary nurse" OR "allied health" OR "basic health worker" OR barefoot OR "bare foot" OR "birth attendant" OR "bridge to health team" OR "care group" OR "case coordinator" OR "child health worker" OR "community health worker" OR chw* OR "close to community provider" OR "community agent" OR "community aide" OR "community based practitioner" OR "community case management" OR "community coordinator" OR "community drug distributor" OR "community health assistant" OR "community health aide" OR "community health agent" OR "community health care provider" OR "community healthcare provider" OR "community health extension worker" OR "community health nurse" OR "community health representative" OR "community health surveyor" OR "community health volunteer" OR "community healthcare worker" OR "community health care worker" OR "community healthcare provider" OR "community health care provider" OR "community health promoter" OR "community health care provider" OR "community liaison" OR "community nutrition worker" OR "community practitioner" OR "community resource person" OR "community surveillance volunteer" OR "community volunteer" OR "community worker" OR "care group" OR "dame health worker" OR "door to door" OR "extension service" OR "extension officer" OR "extension staff" OR "extension worker" OR "family planning agent" OR "family advocate" OR "family support worker" OR "family welfare assistant" OR "family welfare worker" OR "field based" OR grassroots OR "grass roots" OR "hard to reach" OR "health activist" OR "health aide" OR "health agent" OR "health care agent" OR "healthcare agent" OR "health assistant" OR "health auxiliar" OR "health care worker" OR "healthcare worker" OR "health coach" OR "health counselor" OR "health development army" OR "health distributor" OR "health education" OR "health extension" OR "health nurse" OR "health officer" OR "health motivator" OR "health outreach" OR "health out reach" OR "health promoter" OR "health promotor" OR "health surveillance assistant" OR "health visitor" OR "health worker" OR "health volunteer" OR "home based care" OR "home care" OR "home health" OR "home service" OR "home visit" OR iccm* OR imci* OR "intake specialist" OR "lady health worker" OR "lay aide" OR "lay aides" OR "lay attendant" OR "lay consultant" OR "lay counselor" OR "lay health advisor" OR "lay health worker" OR lhw* OR "lay visitor" OR "lay worker" OR "lead mother" OR "link worker" OR "malaria agent" OR "child health worker" OR "child health worker" OR "medical assistant" OR midwife* OR "mid wife" OR "mid wifes" OR midwive* OR "mid wive" OR "mobile clinic team" OR "mother coordinator" OR "mother leader" OR navigator* OR "nutrition agent" OR "nutrition counselor" OR "outreach advocate" OR "outreach case manager" OR "outreach educator" OR "outreach worker" OR "out reach advocate" OR "out reach case manager" OR "out reach educator" OR "out reach worker" OR "parent liaison" OR "peer advisor" OR "peer counselor" OR "peer educator" OR "peer health advisor" OR "peer leader" OR "peer supporter" OR "promotora" OR "rural health auxiliar" OR "support worker" OR "traditional birth attendant" OR "village health worker" OR vhw* OR "village drug kit manager" OR "village health" OR "voluntary health worker" OR "voluntary worker" OR volunteer*

1. **Mhealth**

android OR app OR apps OR "cell phone" OR "cell phones" OR "cellular phone" OR "cellular phones" OR desktop* OR "desk top" OR "digital health" OR "digital diagnostic device" OR "distance consult" OR "distance counselling" OR "distant consult" OR "e diagnosis" OR "e coach" OR "e coaches" OR "e coaching" OR econsult* OR "e consult" OR "e consults" OR ediagnos* OR ehealth* OR "e health" OR exergam* OR facebook OR "face book" OR "feature phone" OR "feature phones" OR game OR games OR gamification OR gaming OR "global positioning system" OR gps OR "health app" OR "health apps" OR "health kiosk" OR "health technology" OR "interactive voice response" OR internet OR ipad OR ipads OR iphone OR "i pad" OR "i pads" OR "i phone" OR laptop* OR "lap top" OR mhapp* OR "mh app" OR "mh apps" OR mhealth OR "m health" OR "mobile app" OR "mobile apps" OR "mobile health" OR "mobile technology" OR "mobile device" OR "mobile devices" OR "mobile phone" OR "mobile phones" OR "palm top" OR "palm tops" OR palmtop* OR "patient portal" OR "patient portals" OR pda OR pdas OR "personal digital assistant" OR "personal electronic health record" OR "personal health record" OR "phone app" OR "phone apps" OR "portable media player" OR "radio frequency identification" OR rfid OR "remote consult" OR "remote consults" OR "remote counselling" OR "satellite phone" OR "satellite phones" OR smartphone* OR "smart phone" OR "smart phones" OR sms OR "social media" OR tablets OR tablet OR "tele app" OR "tele apps" OR "tele care" OR "tele consult" OR "tele counselling" OR "tele diagnosis" OR "tele health" OR "tele medicine" OR "tele monitoring" OR "tele nursing" OR telecare* OR teleconsult* OR telecounsel* OR telediagnos* OR telehealth OR telemedic* OR telemonitor* OR telenursing* OR "telephone app" OR "telephone apps" OR "text messaging" OR wearable* OR "web portal" OR webportal* OR whatsapp* OR "whats app" OR "world wide web" OR worldwideweb OR www OR nintendo OR twitter OR instagram OR "x box" OR xbox OR "smart watch" OR "smart watches"

**Results: 7 Studies**

## Ebsco/CINAHL (255)

| **#** | **Query** | **Results** |
| --- | --- | --- |
| **S5** | S1 AND S2 AND S3 AND S4 | **255** |
| **S4** | MH "Africa South of the Sahara+" OR MH "Africa, Central+" OR MH "Africa, Eastern+" OR MH "Africa, Southern+" OR MH "Africa, Western+" OR TI("angol*" OR "benin*" OR "botswan*" OR "burkina faso*" OR "burundi*" OR "cabo verd*" OR "cameroon*" OR "cape verd*" OR "central african republic*" OR "chad*" OR "comoros*" OR "congo*" OR "cote d ivoire*" OR "cote diIvoire*" OR "djibouti*" OR "equatorial guinea*" OR "eritrea*" OR "eswatini*" OR "ethiopia*" OR "gabon*" OR "gambia*" OR "ghana" OR "ghanes*" OR "guinea*" OR "ivory coast*" OR "kenya*" OR "lesotho*" OR "liberia*" OR "madagasca*" OR "malawi*" OR "mali" OR "malines*" OR "mauritania*" OR "mauriti*" OR "mozambiq*" OR "namibia*" OR "niger" OR "nigeria*" OR "rhodesia*" OR "ruand*" OR "rwand*" OR "sao tome and principe*" OR "sao tome principe*" OR "senegal*" OR "seychell*" OR "sierra leone*" OR "somalia*" OR "south africa*" OR "rio muni" OR "subsaharan africa*" OR "sub saharan africa*" OR "subsahara africa*" OR "sub sahara africa*" OR "sudan*" OR "swaziland*" OR "swazi land*" OR "tanzan*" OR "togo" OR "togoles*" OR "ugand*" OR "zambia*" OR "zimbabw*" OR "central africa*" OR "east africa*" OR "eastern africa*" OR "southern africa*" OR "west africa*" OR "western africa*" OR "africa south of the sahara") OR AB("angol*" OR "benin*" OR "botswan*" OR "burkina faso*" OR "burundi*" OR "cabo verd*" OR "cameroon*" OR "cape verd*" OR "central african republic*" OR "chad*" OR "comoros*" OR "congo*" OR "cote d ivoire*" OR "cote diIvoire*" OR "djibouti*" OR "equatorial guinea*" OR "eritrea*" OR "eswatini*" OR "ethiopia*" OR "gabon*" OR "gambia*" OR "ghana" OR "ghanes*" OR "guinea*" OR "ivory coast*" OR "kenya*" OR "lesotho*" OR "liberia*" OR "madagasca*" OR "malawi*" OR "mali" OR "malines*" OR "mauritania*" OR "mauriti*" OR "mozambiq*" OR "namibia*" OR "niger" OR "nigeria*" OR "rhodesia*" OR "ruand*" OR "rwand*" OR "sao tome and principe*" OR "sao tome principe*" OR "senegal*" OR "seychell*" OR "sierra leone*" OR "somalia*" OR "south africa*" OR "rio muni" OR "subsaharan africa*" OR "sub saharan africa*" OR "subsahara africa*" OR "sub sahara africa*" OR "sudan*" OR "swaziland*" OR "swazi land*" OR "tanzan*" OR "togo" OR "togoles*" OR "ugand*" OR "zambia*" OR "zimbabw*" OR "central africa*" OR "east africa*" OR "eastern africa*" OR "southern africa*" OR "west africa*" OR "western africa*" OR "africa south of the sahara") OR KW("angol*" OR "benin*" OR "botswan*" OR "burkina faso*" OR "burundi*" OR "cabo verd*" OR "cameroon*" OR "cape verd*" OR "central african republic*" OR "chad*" OR "comoros*" OR "congo*" OR "cote d ivoire*" OR "cote diIvoire*" OR "djibouti*" OR "equatorial guinea*" OR "eritrea*" OR "eswatini*" OR "ethiopia*" OR "gabon*" OR "gambia*" OR "ghana" OR "ghanes*" OR "guinea*" OR "ivory coast*" OR "kenya*" OR "lesotho*" OR "liberia*" OR "madagasca*" OR "malawi*" OR "mali" OR "malines*" OR "mauritania*" OR "mauriti*" OR "mozambiq*" OR "namibia*" OR "niger" OR "nigeria*" OR "rhodesia*" OR "ruand*" OR "rwand*" OR "sao tome and principe*" OR "sao tome principe*" OR "senegal*" OR "seychell*" OR "sierra leone*" OR "somalia*" OR "south africa*" OR "rio muni" OR "subsaharan africa*" OR "sub saharan africa*" OR "subsahara africa*" OR "sub sahara africa*" OR "sudan*" OR "swaziland*" OR "swazi land*" OR "tanzan*" OR "togo" OR "togoles*" OR "ugand*" OR "zambia*" OR "zimbabw*" OR "central africa*" OR "east africa*" OR "eastern africa*" OR "southern africa*" OR "west africa*" OR "western africa*" OR "africa south of the sahara") OR AF("angol*" OR "benin*" OR "botswan*" OR "burkina faso*" OR "burundi*" OR "cabo verd*" OR "cameroon*" OR "cape verd*" OR "central african republic*" OR "chad*" OR "comoros*" OR "congo*" OR "cote d ivoire*" OR "cote diIvoire*" OR "djibouti*" OR "equatorial guinea*" OR "eritrea*" OR "eswatini*" OR "ethiopia*" OR "gabon*" OR "gambia*" OR "ghana" OR "ghanes*" OR "guinea*" OR "ivory coast*" OR "kenya*" OR "lesotho*" OR "liberia*" OR "madagasca*" OR "malawi*" OR "mali" OR "malines*" OR "mauritania*" OR "mauriti*" OR "mozambiq*" OR "namibia*" OR "niger" OR "nigeria*" OR "rhodesia*" OR "ruand*" OR "rwand*" OR "sao tome and principe*" OR "sao tome principe*" OR "senegal*" OR "seychell*" OR "sierra leone*" OR "somalia*" OR "south africa*" OR "rio muni" OR "subsaharan africa*" OR "sub saharan africa*" OR "subsahara africa*" OR "sub sahara africa*" OR "sudan*" OR "swaziland*" OR "swazi land*" OR "tanzan*" OR "togo" OR "togoles*" OR "ugand*" OR "zambia*" OR "zimbabw*" OR "central africa*" OR "east africa*" OR "eastern africa*" OR "southern africa*" OR "west africa*" OR "western africa*" OR "africa south of the sahara") | **130,969** |
| **S3** | MH "Text Messaging" OR MH "Cellular Phone" OR MH "Smartphone" OR MH "Voice Mail" OR MH "Computers, Portable+" OR MH "Microcomputers" OR MH "Minicomputers" OR MH "Patient Portals" OR MH "Mobile Applications" OR MH "Digital Health" OR MH "Telehealth" OR MH "Wearable Sensors" OR MH "Games+" OR MH "Global Positioning System" OR MH "Instant Messaging" OR MH "Telecommunications" OR MH "Electronic Bulletin Boards" OR MH "Internet+" OR MH "Email" OR MH "Social Media+" OR MH "Teleconferencing" OR MH "Telehealth" OR MH "Telemedicine" OR MH "Telenursing" OR MH "Telephone" OR MH "Webcasts+" OR MH "Videoconferencing+" OR MH "Wireless Communications" OR MH "Mobile Applications" OR MH "Web Browsers" OR TI("android" OR "app" OR "apps" OR "cell phone*" OR "cellular phone*" OR "desktop*" OR "desk top*" OR "digital health" OR "digital diagnostic device*" OR "distance consult*" OR "distance counsel*" OR "distant consult*" OR "e diagnos*" OR "e coach*" OR "econsult*" OR "e consult*" OR "ediagnos*" OR "ehealth*" OR "e health*" OR "exergam*" OR "facebook" OR "face book" OR "feature phone*" OR "game" OR "games" OR "gamification" OR "gaming" OR "global positioning system*" OR "gps" OR "health app*" OR "health kiosk*" OR "health technolog*" OR "interactive voice response*" OR "internet*" OR "ipad" OR "ipads" OR "iphone*" OR "i pad" OR "i pads" OR "i phone*" OR "laptop*" OR "lap top*" OR "mhapp*" OR "mh app*" OR "mhealth*" OR "m health*" OR "mobile app*" OR "mobile health*" OR "mobile technolog*" OR "mobile device*" OR "mobile phone*" OR "palm top*" OR "palmtop*" OR "patient portal*" OR "pda" OR "pdas" OR "personal digital assistant*" OR "personal electronic health record*" OR "personal health record*" OR "phone app*" OR "portable media player*" OR "radio frequency identification*" OR "rfid*" OR "remote consult*" OR "remote counsel*" OR "satellite phone*" OR "serious gam*" OR "smartphone*" OR "smart phone*" OR "smart phone*" OR "sms" OR "social media*" OR "tablets" OR "tablet" OR "tele app*" OR "tele care" OR "tele consult*" OR "tele counsel*" OR "tele diagnos*" OR "tele health" OR "tele medic*" OR "tele monitor*" OR "tele nursing" OR "telecare" OR "teleconsult*" OR "telecounsel*" OR "telediagnos*" OR "telehealth*" OR "telemedic*" OR "telemonitor*" OR "telenursing" OR "telephone app*" OR "text messag*" OR "wearable*" OR "web portal*" OR "webportal*" OR "whatsapp*" OR "whats app*" OR "world wide web" OR "worldwideweb" OR "www" OR "nintendo" OR "twitter" OR "instagram" OR "x box" OR "xbox" OR "smart watch*") OR AB("android" OR "app" OR "apps" OR "cell phone*" OR "cellular phone*" OR "desktop*" OR "desk top*" OR "digital health" OR "digital diagnostic device*" OR "distance consult*" OR "distance counsel*" OR "distant consult*" OR "e diagnos*" OR "e coach*" OR "econsult*" OR "e consult*" OR "ediagnos*" OR "ehealth*" OR "e health*" OR "exergam*" OR "facebook" OR "face book" OR "feature phone*" OR "game" OR "games" OR "gamification" OR "gaming" OR "global positioning system*" OR "gps" OR "health app*" OR "health kiosk*" OR "health technolog*" OR "interactive voice response*" OR "internet*" OR "ipad" OR "ipads" OR "iphone*" OR "i pad" OR "i pads" OR "i phone*" OR "laptop*" OR "lap top*" OR "mhapp*" OR "mh app*" OR "mhealth*" OR "m health*" OR "mobile app*" OR "mobile health*" OR "mobile technolog*" OR "mobile device*" OR "mobile phone*" OR "palm top*" OR "palmtop*" OR "patient portal*" OR "pda" OR "pdas" OR "personal digital assistant*" OR "personal electronic health record*" OR "personal health record*" OR "phone app*" OR "portable media player*" OR "radio frequency identification*" OR "rfid*" OR "remote consult*" OR "remote counsel*" OR "satellite phone*" OR "serious gam*" OR "smartphone*" OR "smart phone*" OR "smart phone*" OR "sms" OR "social media*" OR "tablets" OR "tablet" OR "tele app*" OR "tele care" OR "tele consult*" OR "tele counsel*" OR "tele diagnos*" OR "tele health" OR "tele medic*" OR "tele monitor*" OR "tele nursing" OR "telecare" OR "teleconsult*" OR "telecounsel*" OR "telediagnos*" OR "telehealth*" OR "telemedic*" OR "telemonitor*" OR "telenursing" OR "telephone app*" OR "text messag*" OR "wearable*" OR "web portal*" OR "webportal*" OR "whatsapp*" OR "whats app*" OR "world wide web" OR "worldwideweb" OR "www" OR "nintendo" OR "twitter" OR "instagram" OR "x box" OR "xbox" OR "smart watch*") OR KW("android" OR "app" OR "apps" OR "cell phone*" OR "cellular phone*" OR "desktop*" OR "desk top*" OR "digital health" OR "digital diagnostic device*" OR "distance consult*" OR "distance counsel*" OR "distant consult*" OR "e diagnos*" OR "e coach*" OR "econsult*" OR "e consult*" OR "ediagnos*" OR "ehealth*" OR "e health*" OR "exergam*" OR "facebook" OR "face book" OR "feature phone*" OR "game" OR "games" OR "gamification" OR "gaming" OR "global positioning system*" OR "gps" OR "health app*" OR "health kiosk*" OR "health technolog*" OR "interactive voice response*" OR "internet*" OR "ipad" OR "ipads" OR "iphone*" OR "i pad" OR "i pads" OR "i phone*" OR "laptop*" OR "lap top*" OR "mhapp*" OR "mh app*" OR "mhealth*" OR "m health*" OR "mobile app*" OR "mobile health*" OR "mobile technolog*" OR "mobile device*" OR "mobile phone*" OR "palm top*" OR "palmtop*" OR "patient portal*" OR "pda" OR "pdas" OR "personal digital assistant*" OR "personal electronic health record*" OR "personal health record*" OR "phone app*" OR "portable media player*" OR "radio frequency identification*" OR "rfid*" OR "remote consult*" OR "remote counsel*" OR "satellite phone*" OR "serious gam*" OR "smartphone*" OR "smart phone*" OR "smart phone*" OR "sms" OR "social media*" OR "tablets" OR "tablet" OR "tele app*" OR "tele care" OR "tele consult*" OR "tele counsel*" OR "tele diagnos*" OR "tele health" OR "tele medic*" OR "tele monitor*" OR "tele nursing" OR "telecare" OR "teleconsult*" OR "telecounsel*" OR "telediagnos*" OR "telehealth*" OR "telemedic*" OR "telemonitor*" OR "telenursing" OR "telephone app*" OR "text messag*" OR "wearable*" OR "web portal*" OR "webportal*" OR "whatsapp*" OR "whats app*" OR "world wide web" OR "worldwideweb" OR "www" OR "nintendo" OR "twitter" OR "instagram" OR "x box" OR "xbox" OR "smart watch*") | **343,670** |
| **S2** | MH "Community Health Workers" OR MH "Midwives+" OR MH "Rural Health Personnel" OR TI("accompagnateur*" OR "accredited social health activist*" OR "asha*" OR "animator*" OR "auxiliary nurse*" OR "allied health*" OR "basic health worker*" OR "barefoot*" OR "bare foot*" OR "birth attendant*" OR "bridge to health team*" OR "care group*" OR "case coordinator*" OR "child health worker*" OR "community health worker*" OR "chw*" OR "close to community provider*" OR "community agent*" OR "community aide*" OR "community based practitioner*" OR "community case management*" OR "community coordinator*" OR "community drug distributor*" OR "community health assistant*" OR "community health aide*" OR "community health agent*" OR "community health care provider*" OR "community healthcare provider*" OR "community health extension worker*" OR "community health nurse*" OR "community health representative*" OR "community health surveyor*" OR "community health volunteer*" OR "community healthcare worker*" OR "community health care worker*" OR "community healthcare provider*" OR "community health care provider*" OR "community health promoter*" OR "community health care provider*" OR "community liaison*" OR "community nutrition worker*" OR "community practitioner*" OR "community resource person*" OR "community surveillance volunteer*" OR "community volunteer*" OR "community worker*" OR "care group*" OR "dame health worker*" OR "door to door*" OR "extension service*" OR "extension officer*" OR "extension staff*" OR "extension worker*" OR "family planning agent*" OR "family advocate*" OR "family support worker*" OR "family welfare assistant*" OR "family welfare worker*" OR "field based*" OR "grassroots*" OR "grass roots*" OR "hard to reach*" OR "health activist*" OR "health aide*" OR "health agent*" OR "health care agent*" OR "healthcare agent*" OR "health assistant*" OR "health auxiliar*" OR "health care worker*" OR "healthcare worker*" OR "health coach*" OR "health counselor*" OR "health development army*" OR "health distributor*" OR "health education*" OR "health extension*" OR "health nurse*" OR "health officer*" OR "health motivator*" OR "health outreach*" OR "health out reach*" OR "health promoter*" OR "health promotor*" OR "health surveillance assistant*" OR "health visitor*" OR "health worker*" OR "health volunteer*" OR "home based care*" OR "home care*" OR "home health*" OR "home service*" OR "home visit*" OR "iccm*" OR "imci*" OR "intake specialist*" OR "lady health worker*" OR "lay aide*" OR "lay attendant*" OR "lay consultant*" OR "lay counselor*" OR "lay health advisor*" OR "lay health worker*" OR "lhw*" OR "lay visitor*" OR "lay worker*" OR "lead mother*" OR "link worker*" OR "malaria agent*" OR "child health worker*" OR "child health worker*" OR "medical assistant*" OR "midwife*" OR "mid wife*" OR "midwive*" OR "mid wive*" OR "mobile clinic team*" OR "mother coordinator*" OR "mother leader*" OR "navigator*" OR "nutrition agent*" OR "nutrition counselor*" OR "outreach advocate*" OR "outreach case manager*" OR "outreach educator*" OR "outreach worker*" OR "out reach advocate*" OR "out reach case manager*" OR "out reach educator*" OR "out reach worker*" OR "parent liaison*" OR "peer advisor*" OR "peer counselor*" OR "peer educator*" OR "peer health advisor*" OR "peer leader*" OR "peer supporter*" OR "promotora*" OR "rural health auxiliar*" OR "support worker*" OR "surveillance volunteer*" OR "traditional birth attendant*" OR "village health volunteer*" OR "village health worker*" OR "vhw*" OR "village drug kit manager*" OR "village health*" OR "village health helper*" OR "voluntary health worker*" OR "voluntary worker*" OR "volunteer*") OR AB("accompagnateur*" OR "accredited social health activist*" OR "asha*" OR "animator*" OR "auxiliary nurse*" OR "allied health*" OR "basic health worker*" OR "barefoot*" OR "bare foot*" OR "birth attendant*" OR "bridge to health team*" OR "care group*" OR "case coordinator*" OR "child health worker*" OR "community health worker*" OR "chw*" OR "close to community provider*" OR "community agent*" OR "community aide*" OR "community based practitioner*" OR "community case management*" OR "community coordinator*" OR "community drug distributor*" OR "community health assistant*" OR "community health aide*" OR "community health agent*" OR "community health care provider*" OR "community healthcare provider*" OR "community health extension worker*" OR "community health nurse*" OR "community health representative*" OR "community health surveyor*" OR "community health volunteer*" OR "community healthcare worker*" OR "community health care worker*" OR "community healthcare provider*" OR "community health care provider*" OR "community health promoter*" OR "community health care provider*" OR "community liaison*" OR "community nutrition worker*" OR "community practitioner*" OR "community resource person*" OR "community surveillance volunteer*" OR "community volunteer*" OR "community worker*" OR "care group*" OR "dame health worker*" OR "door to door*" OR "extension service*" OR "extension officer*" OR "extension staff*" OR "extension worker*" OR "family planning agent*" OR "family advocate*" OR "family support worker*" OR "family welfare assistant*" OR "family welfare worker*" OR "field based*" OR "grassroots*" OR "grass roots*" OR "hard to reach*" OR "health activist*" OR "health aide*" OR "health agent*" OR "health care agent*" OR "healthcare agent*" OR "health assistant*" OR "health auxiliar*" OR "health care worker*" OR "healthcare worker*" OR "health coach*" OR "health counselor*" OR "health development army*" OR "health distributor*" OR "health education*" OR "health extension*" OR "health nurse*" OR "health officer*" OR "health motivator*" OR "health outreach*" OR "health out reach*" OR "health promoter*" OR "health promotor*" OR "health surveillance assistant*" OR "health visitor*" OR "health worker*" OR "health volunteer*" OR "home based care*" OR "home care*" OR "home health*" OR "home service*" OR "home visit*" OR "iccm*" OR "imci*" OR "intake specialist*" OR "lady health worker*" OR "lay aide*" OR "lay attendant*" OR "lay consultant*" OR "lay counselor*" OR "lay health advisor*" OR "lay health worker*" OR "lhw*" OR "lay visitor*" OR "lay worker*" OR "lead mother*" OR "link worker*" OR "malaria agent*" OR "child health worker*" OR "child health worker*" OR "medical assistant*" OR "midwife*" OR "mid wife*" OR "midwive*" OR "mid wive*" OR "mobile clinic team*" OR "mother coordinator*" OR "mother leader*" OR "navigator*" OR "nutrition agent*" OR "nutrition counselor*" OR "outreach advocate*" OR "outreach case manager*" OR "outreach educator*" OR "outreach worker*" OR "out reach advocate*" OR "out reach case manager*" OR "out reach educator*" OR "out reach worker*" OR "parent liaison*" OR "peer advisor*" OR "peer counselor*" OR "peer educator*" OR "peer health advisor*" OR "peer leader*" OR "peer supporter*" OR "promotora*" OR "rural health auxiliar*" OR "support worker*" OR "surveillance volunteer*" OR "traditional birth attendant*" OR "village health volunteer*" OR "village health worker*" OR "vhw*" OR "village drug kit manager*" OR "village health*" OR "village health helper*" OR "voluntary health worker*" OR "voluntary worker*" OR "volunteer*") OR KW("accompagnateur*" OR "accredited social health activist*" OR "asha*" OR "animator*" OR "auxiliary nurse*" OR "allied health*" OR "basic health worker*" OR "barefoot*" OR "bare foot*" OR "birth attendant*" OR "bridge to health team*" OR "care group*" OR "case coordinator*" OR "child health worker*" OR "community health worker*" OR "chw*" OR "close to community provider*" OR "community agent*" OR "community aide*" OR "community based practitioner*" OR "community case management*" OR "community coordinator*" OR "community drug distributor*" OR "community health assistant*" OR "community health aide*" OR "community health agent*" OR "community health care provider*" OR "community healthcare provider*" OR "community health extension worker*" OR "community health nurse*" OR "community health representative*" OR "community health surveyor*" OR "community health volunteer*" OR "community healthcare worker*" OR "community health care worker*" OR "community healthcare provider*" OR "community health care provider*" OR "community health promoter*" OR "community health care provider*" OR "community liaison*" OR "community nutrition worker*" OR "community practitioner*" OR "community resource person*" OR "community surveillance volunteer*" OR "community volunteer*" OR "community worker*" OR "care group*" OR "dame health worker*" OR "door to door*" OR "extension service*" OR "extension officer*" OR "extension staff*" OR "extension worker*" OR "family planning agent*" OR "family advocate*" OR "family support worker*" OR "family welfare assistant*" OR "family welfare worker*" OR "field based*" OR "grassroots*" OR "grass roots*" OR "hard to reach*" OR "health activist*" OR "health aide*" OR "health agent*" OR "health care agent*" OR "healthcare agent*" OR "health assistant*" OR "health auxiliar*" OR "health care worker*" OR "healthcare worker*" OR "health coach*" OR "health counselor*" OR "health development army*" OR "health distributor*" OR "health education*" OR "health extension*" OR "health nurse*" OR "health officer*" OR "health motivator*" OR "health outreach*" OR "health out reach*" OR "health promoter*" OR "health promotor*" OR "health surveillance assistant*" OR "health visitor*" OR "health worker*" OR "health volunteer*" OR "home based care*" OR "home care*" OR "home health*" OR "home service*" OR "home visit*" OR "iccm*" OR "imci*" OR "intake specialist*" OR "lady health worker*" OR "lay aide*" OR "lay attendant*" OR "lay consultant*" OR "lay counselor*" OR "lay health advisor*" OR "lay health worker*" OR "lhw*" OR "lay visitor*" OR "lay worker*" OR "lead mother*" OR "link worker*" OR "malaria agent*" OR "child health worker*" OR "child health worker*" OR "medical assistant*" OR "midwife*" OR "mid wife*" OR "midwive*" OR "mid wive*" OR "mobile clinic team*" OR "mother coordinator*" OR "mother leader*" OR "navigator*" OR "nutrition agent*" OR "nutrition counselor*" OR "outreach advocate*" OR "outreach case manager*" OR "outreach educator*" OR "outreach worker*" OR "out reach advocate*" OR "out reach case manager*" OR "out reach educator*" OR "out reach worker*" OR "parent liaison*" OR "peer advisor*" OR "peer counselor*" OR "peer educator*" OR "peer health advisor*" OR "peer leader*" OR "peer supporter*" OR "promotora*" OR "rural health auxiliar*" OR "support worker*" OR "surveillance volunteer*" OR "traditional birth attendant*" OR "village health volunteer*" OR "village health worker*" OR "vhw*" OR "village drug kit manager*" OR "village health*" OR "village health helper*" OR "voluntary health worker*" OR "voluntary worker*" OR "volunteer*") | **210,699** |
| **S1** | MH "Postnatal Period+" OR MH "Pregnancy" OR MH "Childbirth+" OR MH "Labor+" OR MH "Labor Presentation+" OR MH "Labor Stages+" OR MH "Perinatal Period" OR MH "Pregnancy, High Risk" OR MH "Pregnancy, Prolonged" OR MH "Pregnancy, Multiple+" OR MH "Reproductive Behavior" OR MH "Obstetric Care+" OR MH "Delivery, Obstetric" OR MH "Intrapartum Care" OR MH "Labor Support" OR MH "Management of Labor" OR MH "Pushing (Childbirth)" OR MH "Postnatal Care+" OR MH "Perinatal Care" OR MH "Prenatal Care" OR MH "Prepregnancy Care" OR MH "Maternal-Child Care" OR MH "Breech Delivery" OR MH "Vaginal Birth" OR MH "Diagnosis, Obstetric" OR MH "Prenatal Diagnosis" OR MH "Midwives+" OR MH "Breast Feeding+" OR MH "Bottle Feeding" OR MH "Reproductive Health" OR MH "Maternal-Child Health" OR MH "Maternal Mortality" OR MH "Motherhood" OR TI("pregnan*" OR "obstetr*" OR "midwive*" OR "midwife*" OR "mid wive*" OR "mid wife*" OR "antenatal" OR "postnatal" OR "postpartum" OR "post natal" OR "post partum" OR "gravid*" OR "partur*" OR "birth*" OR "neonatal*" OR "neo natal*" OR "lactat*" OR "puerper*" OR "labor" OR "labour" OR "term birth" OR "prenatal*" OR "perinatal*" OR "child birth*" OR "childbirth*" OR "newborn*" OR "breastfe*" OR "breast fe*" OR "bottle fe*" OR "reproductive health*" OR ("maternal" N5 ("health*" OR "care*" OR "welfare*" OR "healthcare*" OR "service*" OR "death*" OR "mortal*")) OR "reproductive health*" OR "reproductive service*" OR "pre natal*" OR "prenatal*" OR "antenatal*" OR "safe motherhood*" OR "safe mother hood" OR "breech*") OR AB("pregnan*" OR "obstetr*" OR "midwive*" OR "midwife*" OR "mid wive*" OR "mid wife*" OR "antenatal" OR "postnatal" OR "postpartum" OR "post natal" OR "post partum" OR "gravid*" OR "partur*" OR "birth*" OR "neonatal*" OR "neo natal*" OR "lactat*" OR "puerper*" OR "labor" OR "labour" OR "term birth" OR "prenatal*" OR "perinatal*" OR "child birth*" OR "childbirth*" OR "newborn*" OR "breastfe*" OR "breast fe*" OR "bottle fe*" OR "reproductive health*" OR ("maternal" N5 ("health*" OR "care*" OR "welfare*" OR "healthcare*" OR "service*" OR "death*" OR "mortal*")) OR "reproductive health*" OR "reproductive service*" OR "pre natal*" OR "prenatal*" OR "antenatal*" OR "safe motherhood*" OR "safe mother hood" OR "breech*") OR KW("pregnan*" OR "obstetr*" OR "midwive*" OR "midwife*" OR "mid wive*" OR "mid wife*" OR "antenatal" OR "postnatal" OR "postpartum" OR "post natal" OR "post partum" OR "gravid*" OR "partur*" OR "birth*" OR "neonatal*" OR "neo natal*" OR "lactat*" OR "puerper*" OR "labor" OR "labour" OR "term birth" OR "prenatal*" OR "perinatal*" OR "child birth*" OR "childbirth*" OR "newborn*" OR "breastfe*" OR "breast fe*" OR "bottle fe*" OR "reproductive health*" OR ("maternal" N5 ("health*" OR "care*" OR "welfare*" OR "healthcare*" OR "service*" OR "death*" OR "mortal*")) OR "reproductive health*" OR "reproductive service*" OR "pre natal*" OR "prenatal*" OR "antenatal*" OR "safe motherhood*" OR "safe mother hood" OR "breech*") | **517,038** |

## Google Scholar query (0)

africa+pregnancy|postnatal|postpartum|birth|labor|perinatal|reproductive|maternal health|maternal mortality|prenatal|antenatal|mother+health worker|barefoot|community|volunteer|grassroots|lay+app|phone|digital|ehealth|game|ipad|laptop|mhapp|mhealth|social media|tablet|whatsapp

Date of search 11/10/22

**Results: 0**
