# Supplementary material for: Evaluating the Adoption of mHealth Technologies by Community Health Workers to Improve the Use of Maternal Health Services in Sub-Saharan Africa: Systematic Review
Source: JMIR Mhealth Uhealth. 2024 Sep 24;12:e55819. doi: 10.2196/55819 (PMC11462100; doi:10.2196/55819)
Supplement: Multimedia Appendix 3 [file mhealth_v12i1e55819_app3.docx]

# MULTIMEDIA Appendix 3 Excluded studies and reasons for exclusion

|  | **Title** | **First Author, Year** | **Journal** | **Volume** | **Issue** | **Reason for exclusion** |
| --- | --- | --- | --- | --- | --- | --- |
| 1 | Sustainability of the Effects and Impacts of Using Digital Technology to Extend Maternal Health Services to Rural and Hard-to-Reach Populations: Experience From Southwest Nigeria [1] | Akeju 2022 | Frontiers in global women's health | 3 |  | mHealth use by other health care workers other than CHWs |
| 2 | Using cell phones to collect postpartum hemorrhage outcome data in rural Ghana [2] | Andreatta 2011 | International journal of gynaecology and obstetrics | 113 | 2 | Outcomes other than ANC use, facility-based births, or PNC use |
| 3 | Experiences of women receiving mhealth-supported antenatal care in the village from community health workers in rural Burkina Faso, Africa [3] | Arnaert 2019 | Digital health | 5 |  | Outcomes other than ANC use, facility-based births, or PNC use |
| 4 | mHealth-Based Health Promotion Intervention to Improve Use of Maternity Care Services Among Women in Rural Southwestern Uganda: Iterative Development Study [4] | Atukunda 2021 | JMIR formative research | 5 | 11 | mHealth use by other health care workers other than CHWs |
| 5 | Usability and feasibility of a mobile health system to provide comprehensive antenatal care in low-income countries: PANDA mHealth pilot study in Madagascar [5] | Benski 2017 | Journal of telemedicine and telecare | 23 | 5 | mHealth use by other health care workers other than CHWs |
| 6 | "Quality of prenatal and maternal care: bridging the know-do gap" (QUALMAT study): an electronic clinical decision support system for rural Sub-Saharan Africa [6] | Blank 2013 | BMC medical informatics and decision making | 13 |  | mHealth use by other health care workers other than CHWs |
| 7 | Tanzania Health Information Technology (T-HIT) System: Pilot Test of a Tablet-Based System to Improve Prevention of Mother-to-Child Transmission of HIV [7] | Bull 2018 | JMIR mHealth and uHealth | 6 | 1 | mHealth use by other health care workers other than CHWs |
| 8 | Standardizing Primary Health Care Referral Data Sets in Nigeria: Practitioners' Survey, Form Reviews, and Profiling of Fast Healthcare Interoperability Resources (FHIR) [8] | Chukwu 2022 | JMIR formative research | 6 | 7 | mHealth use by other health care workers other than CHWs |
| 9 | Community-based maternal, newborn, and child health surveillance: perceptions and attitudes of local stakeholders towards using mobile phone by village health volunteers in the Kenge Health Zone, Democratic Republic of Congo [9] | Diese 2018 | BMC public health | 18 | 1 | Outcomes other than ANC use, facility-based births, or PNC use |
| 10 | What Are the Contextual Enablers and Impacts of Using Digital Technology to Extend Maternal and Child Health Services to Rural Areas? Findings of a Qualitative Study From Nigeria [10] | Ebenso 2021 | Frontiers in global women's health | 2 |  | mHealth use by other health care workers other than CHWs |
| 11 | Understanding the introduction and use of a mobile devices supported health information system in Nigeria [11] | Ezenwa 2014 | Electronic Journal of Information Systems in Developing Countries | 62 | 1 | mHealth use by other health care workers other than CHWs |
| 12 | Effectiveness of a Lay Counselor-Led Combination Intervention for Retention of Mothers and Infants in HIV Care: A Randomized Trial in Kenya [12] | Fayorsey 2019 | Journal of acquired immune deficiency syndromes | 80 | 1 | Outcomes other than ANC use, facility-based births, or PNC use |
| 13 | Breastfeeding Interpersonal Communication, Mobile Phone Support, and Mass Media Messaging Increase Exclusive Breastfeeding at 6 and 24 Weeks Among Clients of Private Health Facilities in Lagos, Nigeria [13] | Flax 2022 | The Journal of nutrition | 152 | 5 | mHealth use by other health care workers other than CHWs |
| 14 | Strengthening the home-to-facility continuum of newborn and child health care through mHealth: Evidence from an intervention in rural Malawi [14] | Fotso 2015 | African Population Studies | 29 | 1 | Outcomes other than ANC use, facility-based births, or PNC use |
| 15 | Community based weighing of newborns and use of mobile phones by village elders in rural settings in Kenya: a decentralised approach to health care provision [15] | Gisore 2012 | BMC pregnancy and childbirth | 12 |  | Outcomes other than ANC use, facility-based births, or PNC use |
| 16 | Assessing the feasibility of mobile phones for follow-up of acutely unwell children presenting to village clinics in rural Northern Malawi [16] | Hardy 2017 | Malawi Medical Journal | 29 | 1 | Outcomes other than ANC use, facility-based births, or PNC use |
| 17 | Evaluation of a mHealth Data Quality Intervention to Improve Documentation of Pregnancy Outcomes by Health Surveillance Assistants in Malawi: A Cluster Randomized Trial [17] | Joos 2016 | PloS one | 11 | 1 | Outcomes other than ANC use, facility-based births, or PNC use |
| 18 | Effects of a community-based data for decision-making intervention on maternal and newborn health care practices in Ethiopia: a dose-response study [18] | Karim 2018 | BMC pregnancy and childbirth | 18 | Suppl 1 | Not an mHealth intervention |
| 19 | Testing mHealth solutions at the last mile: insights from a study of technology-assisted community health referrals in rural Kenya [19] | Karlyn 2020 | mHealth | 6 |  | Outcomes other than ANC use, facility-based births, or PNC use |
| 20 | Assessment of mobile health technology for maternal and child health services in rural Upper West Region of Ghana [20] | Laar 2019 | Public health | 168 |  | mHealth use by other health care workers other than CHWs |
| 21 | Meeting community health worker needs for maternal health care service delivery using appropriate mobile technologies in Ethiopia [21] | Little 2013 | PloS one | 8 | 10 | Facility-based mHealth tools |
| 22 | Mobile phones as a health communication tool to improve skilled attendance at delivery in Zanzibar: A cluster-randomised controlled trial [22] | Lund 2012 | BJOG: An International Journal of Obstetrics and Gynaecology | 119 | 10 | mHealth use by other health care workers other than CHWs |
| 23 | Assessment of the quality of antenatal care services provided by health workers using a mobile phone decision support application in northern Nigeria: a pre/post-intervention study [23] | McNabb 2015 | PloS one | 10 | 5 | Outcomes other than ANC use, facility-based births, or PNC use |
| 24 | Health workers' experiences, barriers, preferences and motivating factors in using mHealth forms in Ethiopia [24] | Medhanyie 2015 | Human resources for health | 13 |  | mHealth use by other health care workers other than CHWs |
| 25 | Mobile health data collection at primary health care in Ethiopia: a feasible challenge [25] | Medhanyie 2015 | Journal of clinical epidemiology | 68 | 1 | Facility-based mHealth tools |
| 26 | Why high tech needs high touch: Supporting continuity of community primary health care [26] | Meyer 2018 | African journal of primary health care & family medicine | 10 | 1 | Outcomes other than ANC use, facility-based births, or PNC use |
| 27 | Visibility in community health work mediated by mobile health systems: A case of Malawi [27] | Namatovu 2019 | Electronic Journal of Information Systems in Developing Countries | 85 | 2 | Outcomes other than ANC use, facility-based births, or PNC use |
| 28 | Effect of a multifaceted intervention on the utilisation of primary health for maternal and child health care in rural Nigeria: a quasi-experimental study [28] | Okonofua 2022 | BMJ open | 12 | 2 | mHealth use by other health care workers other than CHWs |
| 29 | Narratives of Women Using a 24-Hour Ride-Hailing Transport System to Increase Access and Utilization of Maternal and Newborn Health Services in Rural Western Kenya: A Qualitative Study [29] | Onono 2019 | The American journal of tropical medicine and hygiene | 101 | 5 | mHealth use by other health care workers other than CHWs |
| 30 | [Acceptability and satisfaction with pregnancy and newborn diagnostic assessment (PANDA) system providing prenatal care in Burkina Faso] [30] | Ouedraogo 2020 | Pan African Medical Journal | 37 |  | mHealth use by other health care workers other than CHWs |
| 31 | Midwives acceptance of mHealth applications in the dissemination of maternal health education in primary healthcare clinics [31] | Ramnund 2021 | Information Development |  |  | mHealth use by other health care workers other than CHWs |
| 32 | A mobile phone-based, community health worker program for referral, follow-up, and service outreach in rural Zambia: Outcomes and overview [32] | Schuttner 2014 | Telemedicine and e-Health | 20 | 8 | Outcomes other than ANC use, facility-based births, or PNC use |
| 33 | Task-shifting of antiretroviral delivery from health care workers to persons living with HIV/AIDS: clinical outcomes of a community-based program in Kenya [33] | Selke 2010 | Journal of acquired immune deficiency syndromes | 55 | 4 | Outcomes other than ANC use, facility-based births, or PNC use |
| 34 | The Effects of a Locally Developed mHealth Intervention on Delivery and Postnatal Care Utilization; A Prospective Controlled Evaluation among Health Centres in Ethiopia [34] | Shiferaw 2016 | PloS one | 11 | 7 | mHealth use by other health care workers other than CHWs |
| 35 | Promising adoption of an electronic clinical decision support system for antenatal and intrapartum care in rural primary healthcare facilities in sub-Saharan Africa: The QUALMAT experience [35] | Sukums 2015 | International journal of medical informatics | 84 | 9 | mHealth use by other health care workers other than CHWs |
| 36 | Health workers' experiences with the Safe Delivery App in West Wollega Zone, Ethiopia: a qualitative study [36] | Thomsen 2019 | Reproductive health | 16 | 1 | Facility-based mHealth tools |
| 37 | Antenatal depression case finding by community health workers in South Africa: feasibility of a mobile phone application [37] | Tsai 2014 | Archives of women's mental health | 17 | 5 | Outcomes other than ANC use, facility-based births, or PNC use |
| 38 | Giving cell phones to pregnant women and improving services may increase primary health facility utilization: a case-control study of a Nigerian project [38] | Oyeyemi 2014 | Reproductive Health | 11 |  | mHealth use by other health care workers other than CHWs |
| 39 | Socio-cultural contextual factors that contribute to the uptake of a mobile health intervention to enhance maternal health care in rural Senegal [39] | MacDonald 2019 | Reproductive Health | 16 |  | Outcomes other than ANC use, facility-based births, or PNC use |
| 40 | Effects of an expanded Uber-like transport system on access to and use of maternal and newborn health services: findings of a prospective cohort study in Homa Bay, Kenya [40] | Onono 2019 | BMJ Global Health | 4 |  | mHealth not used by CHWs to increase outcomes of interest |
| 41 | Boosting antenatal care attendance and number of hospital deliveries among pregnant women in rural communities: a community initiative in Ghana based on mobile phones applications and portable ultrasound scans [41] | Amoah 2016 | BMC Pregnancy and Childbirth | 16 |  | mHealth not used by CHWs to increase outcomes of interest |
| 42 | 'The phone is my boss and my helper' - A gender analysis of an mHealth intervention with Health Extension Workers in Southern Ethiopia [42] | Steege 2018 | Journal of Public Health | Supp. 2 |  | Role of mHealth not clear |
| 43 | Multistakeholder Perspectives on Maternal Text Messaging Intervention in Uganda: Qualitative Study [43] | Ilozumba 2018 | JMIR mHealth and uHealth. 2018 | 5 | 5 | mHealth not used by CHWs to increase outcomes of interest |
| 44 | Can a community health worker administered postnatal checklist increase health-seeking behaviors and knowledge?: evidence from a randomized trial with a private maternity facility in Kiambu County, Kenya [44] | McConnell 2016 | BMC Pregnancy and Childbirth | 16 |  | mHealth not used by CHWs to increase outcomes of interest |

ANC: antenatal care; CHW: community health worker; mHealth: mobile health; PNC: postnatal care.

**References**

1. Akeju D, Okusanya B, Okunade K, Ajepe A, Allsop MJ, Ebenso B. Sustainability of the Effects and Impacts of Using Digital Technology to Extend Maternal Health Services to Rural and Hard-to-Reach Populations: Experience From Southwest Nigeria. Frontiers in global women's health. 2022;3:696529. doi: 10.3389/fgwh.2022.696529.

2. Andreatta P, Debpuur D, Danquah A, Perosky J. Using cell phones to collect postpartum hemorrhage outcome data in rural Ghana. International journal of gynaecology and obstetrics: the official organ of the International Federation of Gynaecology and Obstetrics. 2011;113(2):148-51. doi: https://dx.doi.org/10.1016/j.ijgo.2010.11.020.

3. Arnaert A, Ponzoni N, Debe Z, Meda MM, Nana NG, Arnaert S. Experiences of women receiving mhealth-supported antenatal care in the village from community health workers in rural Burkina Faso, Africa. Digital health. 2019;5:2055207619892756. doi: https://dx.doi.org/10.1177/2055207619892756.

4. Atukunda EC, Matthews LT, Musiimenta A, Mugyenyi GR, Mugisha S, Ware NC, et al. mHealth-Based Health Promotion Intervention to Improve Use of Maternity Care Services Among Women in Rural Southwestern Uganda: Iterative Development Study. JMIR formative research. 2021;5(11):e29214. doi: https://dx.doi.org/10.2196/29214.

5. Benski AC, Stancanelli G, Scaringella S, Herinainasolo JL, Jinoro J, Vassilakos P, et al. Usability and feasibility of a mobile health system to provide comprehensive antenatal care in low-income countries: PANDA mHealth pilot study in Madagascar. Journal of telemedicine and telecare. 2017;23(5):536-43. doi: https://dx.doi.org/10.1177/1357633X16653540.

6. Blank A, Prytherch H, Kaltschmidt J, Krings A, Sukums F, Mensah N, et al. "Quality of prenatal and maternal care: bridging the know-do gap" (QUALMAT study): an electronic clinical decision support system for rural Sub-Saharan Africa. BMC medical informatics and decision making. 2013;13:44. doi: https://dx.doi.org/10.1186/1472-6947-13-44.

7. Bull S, Thomas DS, Nyanza EC, Ngallaba SE. Tanzania Health Information Technology (T-HIT) System: Pilot Test of a Tablet-Based System to Improve Prevention of Mother-to-Child Transmission of HIV. JMIR mHealth and uHealth. 2018;6(1):e16. doi: https://dx.doi.org/10.2196/mhealth.8513.

8. Chukwu E, Garg L, Obande-Ogbuinya N, Chattu VK. Standardizing Primary Health Care Referral Data Sets in Nigeria: Practitioners' Survey, Form Reviews, and Profiling of Fast Healthcare Interoperability Resources (FHIR). JMIR formative research. 2022;6(7):e28510. doi: https://dx.doi.org/10.2196/28510.

9. Diese M, Kalonji A, Izale B, Villeneuve S, Kintaudi NM, Clarysse G, et al. Community-based maternal, newborn, and child health surveillance: perceptions and attitudes of local stakeholders towards using mobile phone by village health volunteers in the Kenge Health Zone, Democratic Republic of Congo. BMC public health. 2018;18(1):316. doi: https://dx.doi.org/10.1186/s12889-018-5186-2.

10. Ebenso B, Okusanya B, Okunade K, Akeju D, Ajepe A, Akaba GO, et al. What Are the Contextual Enablers and Impacts of Using Digital Technology to Extend Maternal and Child Health Services to Rural Areas? Findings of a Qualitative Study From Nigeria. Frontiers in global women's health. 2021;2:670494. doi: https://dx.doi.org/10.3389/fgwh.2021.670494.

11. Ezenwa C, Brooks L. Understanding the introduction and use of a mobile devicesupported health information system in Nigeria. Electronic Journal of Information Systems in Developing Countries. 2014;62(1):1-20. doi: 10.1002/j.1681-4835.2014.tb00445.x.

12. Fayorsey RN, Wang C, Chege D, Reidy W, Syengo M, Owino SO, et al. Effectiveness of a Lay Counselor-Led Combination Intervention for Retention of Mothers and Infants in HIV Care: A Randomized Trial in Kenya. Journal of acquired immune deficiency syndromes (1999). 2019;80(1):56-63. doi: https://dx.doi.org/10.1097/QAI.0000000000001882.

13. Flax VL, Ipadeola A, Schnefke CH, Ralph-Opara U, Adeola O, Edwards S, et al. Breastfeeding Interpersonal Communication, Mobile Phone Support, and Mass Media Messaging Increase Exclusive Breastfeeding at 6 and 24 Weeks Among Clients of Private Health Facilities in Lagos, Nigeria. The Journal of nutrition. 2022;152(5):1316-26. doi: https://dx.doi.org/10.1093/jn/nxab450.

14. Fotso JC, Bellhouse L, Vesel L, Jezman Z. Strengthening the home-to-facility continuum of newborn and child health care through mhealth: Evidence from an intervention in rural Malawi. Etude de la Population Africaine. 2015;29(1):1663-82. doi: 10.11564/29-1-717.

15. Gisore P, Shipala E, Otieno K, Rono B, Marete I, Tenge C, et al. Community based weighing of newborns and use of mobile phones by village elders in rural settings in Kenya: a decentralised approach to health care provision. BMC pregnancy and childbirth. 2012;12:15. doi: https://dx.doi.org/10.1186/1471-2393-12-15.

16. Hardy V, Hsieh J, Chirambo B, Wu TSJ, O’Donoghue J, Muula AS, et al. Assessing the feasibility of mobile phones for follow-up of acutely unwell children presenting to village clinics in rural Northern Malawi. Malawi Medical Journal. 2017;29(1):53-4. doi: 10.4314/mmj.v29i1.10.

17. Joos O, Silva R, Amouzou A, Moulton LH, Perin J, Bryce J, et al. Evaluation of a mHealth Data Quality Intervention to Improve Documentation of Pregnancy Outcomes by Health Surveillance Assistants in Malawi: A Cluster Randomized Trial. PloS one. 2016;11(1):e0145238. doi: https://dx.doi.org/10.1371/journal.pone.0145238.

18. Karim AM, Fesseha Zemichael N, Shigute T, Emaway Altaye D, Dagnew S, Solomon F, et al. Effects of a community-based data for decision-making intervention on maternal and newborn health care practices in Ethiopia: a dose-response study. BMC pregnancy and childbirth. 2018;18(Suppl 1):359. doi: https://dx.doi.org/10.1186/s12884-018-1976-x.

19. Karlyn A, Odindo S, Onyango R, Mbindyo C, Mberi T, Too G, et al. Testing mHealth solutions at the last mile: insights from a study of technology-assisted community health referrals in rural Kenya. mHealth. 2020;6:43. doi: https://dx.doi.org/10.21037/mhealth-19-261.

20. Laar AS, Bekyieriya E, Isang S, Baguune B. Assessment of mobile health technology for maternal and child health services in rural Upper West Region of Ghana. Public health. 2019;168:1-8. doi: https://dx.doi.org/10.1016/j.puhe.2018.11.014.

21. Little A, Medhanyie A, Yebyo H, Spigt M, Dinant G-J, Blanco R. Meeting community health worker needs for maternal health care service delivery using appropriate mobile technologies in Ethiopia. PloS one. 2013;8(10):e77563. doi: https://dx.doi.org/10.1371/journal.pone.0077563.

22. Lund S, Hemed M, Nielsen BB, Said A, Said K, Makungu MH, et al. Mobile phones as a health communication tool to improve skilled attendance at delivery in Zanzibar: A cluster-randomised controlled trial. BJOG: An International Journal of Obstetrics and Gynaecology. 2012;119(10):1256-64. doi: 10.1111/j.1471-0528.2012.03413.x.

23. McNabb M, Chukwu E, Ojo O, Shekhar N, Gill CJ, Salami H, et al. Assessment of the quality of antenatal care services provided by health workers using a mobile phone decision support application in northern Nigeria: a pre/post-intervention study. PloS one. 2015;10(5):e0123940. doi: https://dx.doi.org/10.1371/journal.pone.0123940.

24. Medhanyie AA, Little A, Yebyo H, Spigt M, Tadesse K, Blanco R, et al. Health workers' experiences, barriers, preferences and motivating factors in using mHealth forms in Ethiopia. Human resources for health. 2015;13:2. doi: https://dx.doi.org/10.1186/1478-4491-13-2.

25. Medhanyie AA, Moser A, Spigt M, Yebyo H, Little A, Dinant G, et al. Mobile health data collection at primary health care in Ethiopia: a feasible challenge. Journal of clinical epidemiology. 2015;68(1):80-6. doi: https://dx.doi.org/10.1016/j.jclinepi.2014.09.006.

26. Meyer ED, Hugo JFM, Marcus TS, Molebatsi R, Komana K. Why high tech needs high touch: Supporting continuity of community primary health care. African journal of primary health care & family medicine. 2018;10(1):e1-e6. doi: https://dx.doi.org/10.4102/phcfm.v10i1.1616.

27. Namatovu E, Kanjo C. Visibility in community health work mediated by mobile health systems: A case of Malawi. Electronic Journal of Information Systems in Developing Countries. 2019;85(2). doi: 10.1002/isd2.12071.

28. Okonofua F, Ntoimo LF, Yaya S, Igboin B, Solanke O, Ekwo C, et al. Effect of a multifaceted intervention on the utilisation of primary health for maternal and child health care in rural Nigeria: a quasi-experimental study. BMJ open. 2022;12(2):e049499. doi: https://dx.doi.org/10.1136/bmjopen-2021-049499.

29. Onono M, Odhiambo GO, Congo O, Waguma LW, Serem T, Owenga MA, et al. Narratives of Women Using a 24-Hour Ride-Hailing Transport System to Increase Access and Utilization of Maternal and Newborn Health Services in Rural Western Kenya: A Qualitative Study. The American journal of tropical medicine and hygiene. 2019;101(5):1000-8. doi: https://dx.doi.org/10.4269/ajtmh.19-0132.

30. Ouedraogo AM, Compaore R, Some A, Dahourou DL, Cisse K, Tougri H, et al. [Acceptability and satisfaction with pregnancy and newborn diagnostic assessment (PANDA) system providing prenatal care in Burkina Faso]. Acceptabilite et satisfaction de l'utilisation du systeme Pregnancy and Newborn Diagnostic Assessment (PANDA) pour les soins prenatals au Burkina Faso. 2020;37:361. doi: https://dx.doi.org/10.11604/pamj.2020.37.361.25167.

31. Ramnund S, Baloyi OB, Nkwanyana NM, Jarvis MA. Midwives’ acceptance of mHealth applications in the dissemination of maternal health education in primary healthcare clinics. Information Development. 2021. doi: 10.1177/02666669211049140.

32. Schuttner L, Sindano N, Theis M, Zue C, Joseph J, Chilengi R, et al. A mobile phone-based, community health worker program for referral, follow-up, and service outreach in rural Zambia: Outcomes and overview. Telemedicine and e-Health. 2014;20(8):721-8. doi: 10.1089/tmj.2013.0240.

33. Selke HM, Kimaiyo S, Sidle JE, Vedanthan R, Tierney WM, Shen C, et al. Task-shifting of antiretroviral delivery from health care workers to persons living with HIV/AIDS: clinical outcomes of a community-based program in Kenya. Journal of acquired immune deficiency syndromes (1999). 2010;55(4):483-90. doi: https://dx.doi.org/10.1097/QAI.0b013e3181eb5edb.

34. Shiferaw S, Spigt M, Tekie M, Abdullah M, Fantahun M, Dinant G-J. The Effects of a Locally Developed mHealth Intervention on Delivery and Postnatal Care Utilization; A Prospective Controlled Evaluation among Health Centres in Ethiopia. PloS one. 2016;11(7):e0158600. doi: https://dx.doi.org/10.1371/journal.pone.0158600.

35. Sukums F, Mensah N, Mpembeni R, Massawe S, Duysburgh E, Williams A, et al. Promising adoption of an electronic clinical decision support system for antenatal and intrapartum care in rural primary healthcare facilities in sub-Saharan Africa: The QUALMAT experience. International journal of medical informatics. 2015;84(9):647-57. doi: https://dx.doi.org/10.1016/j.ijmedinf.2015.05.002.

36. Thomsen CF, Barrie AMF, Boas IM, Lund S, Sorensen BL, Oljira FG, et al. Health workers' experiences with the Safe Delivery App in West Wollega Zone, Ethiopia: a qualitative study. Reproductive health. 2019;16(1):50. doi: https://dx.doi.org/10.1186/s12978-019-0725-6.

37. Tsai AC, Tomlinson M, Dewing S, le Roux IM, Harwood JM, Chopra M, et al. Antenatal depression case finding by community health workers in South Africa: feasibility of a mobile phone application. Archives of women's mental health. 2014;17(5):423-31. doi: https://dx.doi.org/10.1007/s00737-014-0426-7.

38. Oyeyemi SO, Wynn R. Giving cell phones to pregnant women and improving services may increase primary health facility utilization: a case-control study of a Nigerian project. Reproductive Health. 2014;11(1):1-16. PMID: 104030407. Language: English. Entry Date: 20140220. Revision Date: 20200708. Publication Type: Journal Article. doi: 10.1186/1742-4755-11-8.

39. MacDonald ME, Diallo GS. Socio-cultural contextual factors that contribute to the uptake of a mobile health intervention to enhance maternal health care in rural Senegal. Reproductive health. 2019;16(1):141. doi: https://dx.doi.org/10.1186/s12978-019-0800-z.

40. Onono MA, Wahome S, Wekesa P, Adhu CK, Waguma LW, Serem T, et al. Effects of an expanded Uber-like transport system on access to and use of maternal and newborn health services: findings of a prospective cohort study in Homa Bay, Kenya. BMJ GLOBAL HEALTH. 2019 MAY;4(3). PMID: WOS:000471834400009. doi: 10.1136/bmjgh-2018-001254.

41. Amoah B, Anto EA, Osei PK, Pieterson K, Crimi A. Boosting antenatal care attendance and number of hospital deliveries among pregnant women in rural communities: a community initiative in Ghana based on mobile phones applications and portable ultrasound scans. BMC pregnancy and childbirth. 2016;16(1):141. doi: https://dx.doi.org/10.1186/s12884-016-0888-x.

42. Steege R, Waldman L, Datiko DG, Kea AZ, Taegtmeyer M, Theobald S. 'The phone is my boss and my helper' - A gender analysis of an mHealth intervention with Health Extension Workers in Southern Ethiopia. Journal of public health (Oxford, England). 2018;40(suppl_2):ii16-ii31. doi: https://dx.doi.org/10.1093/pubmed/fdy199.

43. Ilozumba O, Dieleman M, Van Belle S, Mukuru M, Bardaji A, Broerse JE. Multistakeholder Perspectives on Maternal Text Messaging Intervention in Uganda: Qualitative Study. JMIR mHealth and uHealth. 2018;6(5):e119. doi: 10.2196/mhealth.9565.

44. McConnell M, Ettenger A, Rothschild CW, Muigai F, Cohen J. Can a community health worker administered postnatal checklist increase health-seeking behaviors and knowledge?: evidence from a randomized trial with a private maternity facility in Kiambu County, Kenya. BMC pregnancy and childbirth. 2016;16(1):136. doi: https://dx.doi.org/10.1186/s12884-016-0914-z.
