# Supplementary material for: Evaluating the Adoption of mHealth Technologies by Community Health Workers to Improve the Use of Maternal Health Services in Sub-Saharan Africa: Systematic Review
Source: JMIR Mhealth Uhealth. 2024 Sep 24;12:e55819. doi: 10.2196/55819 (PMC11462100; doi:10.2196/55819)
Supplement: Multimedia Appendix 4 [file mhealth_v12i1e55819_app4.docx]

**MULTIMEDIA APPENDIX 4 RISK-OF-BIAS ASSESSMENT**

Risk-of-bias assessment of qualitative studies using the Mixed Methods Assessment Tool.

| Study, year | Screening question 1 (for all types): are there clear research questions? | Screening question 2 (for all types): do the collected data allow to address the research questions? | Screening question 1 for qualitative studies: is the qualitative approach appropriate to answer the research question? | Screening question 2 for qualitative studies: are the qualitative data collection methods adequate to address the research question? | Screening question 3 for qualitative studies: are the findings adequately derived from the data? | Screening question 4 for qualitative studies: is the interpretation of results sufficiently substantiated by data? | Screening question 5 for qualitative studies: is there coherence between qualitative data sources, collection, analysis, and interpretation? | Comments |
| --- | --- | --- | --- | --- | --- | --- | --- | --- |
| Webber et al [1], 2020 | Yes | Yes | Yes | Can’t tell | Yes | Yes | Yes | Unclear risk of bias: reasons for choosing the sampling method are not described |
| Ayiasi et al [2], 2015 | Yes | Yes | Yes | Yes | Yes | Yes | Yes | Low risk of bias |
| Musabyimana et al [3], 2018 | Yes | Yes | Yes | Yes | Yes | Yes | Yes | Low risk of bias |
| Mwendwa [4], 2016 | Yes | Yes | Yes | Yes | Yes | Yes | Yes | Low risk of bias |

Risk-of-bias assessment of quantitative randomized controlled trials using the Mixed Methods Assessment Tool.

| Study, year | Screening question 1 (for all types): are there clear research questions? | Screening question 2 (for all types): do the collected data allow to address the research questions? | Screening question 1 for quantitative randomized controlled trials: is randomization appropriately performed? | Screening question 2 for quantitative randomized controlled trials: are the groups comparable at baseline? | Screening question 3 for quantitative randomized controlled trials: are there complete outcome data? | Screening question 4 for quantitative randomized controlled trials: are outcome assessors blinded to the intervention provided? | Screening question 5 for quantitative randomized controlled trials: did the participants adhere to the assigned intervention? | Comments |
| --- | --- | --- | --- | --- | --- | --- | --- | --- |
| Sevene et al [5], 2020 | Yes | Yes | Yes | Yes | Yes | Yes | Can’t tell | Unclear risk of bias: the authors commented that participants may have crossed over between the intervention and control sites, but this was neither quantified nor addressed by the study design |
| Ayiasi et al [6], 2016 | Yes | Yes | Yes | No | Yes | No | No | High risk of bias: household and individual characteristics were not comparable (assessors were aware of control and intervention sites) |
| Hackett et al [7], 2018 | Yes | Yes | Yes | Yes | Yes | No | Yes | High risk of bias: no blinding |
| Atnafu et al [8], 2017 | Yes | Yes | Yes | Yes | Yes | No | Can’t tell | High risk of bias: no blinding; potential crossover effect, but the authors did not provide an explanation for this in the manuscript |

Risk-of-bias assessment of quantitative nonrandomized studies using the Mixed Methods Assessment Tool.

| Study, year | Screening question 1 (for all types): are there clear research questions? | Screening question 2 (for all types): do the collected data allow to address the research questions? | Screening question 1 for quantitative nonrandomized studies: are the participants representative of the target population? | Screening question 2 for quantitative nonrandomized studies: are measurements appropriate regarding both the outcome and intervention (or exposure)? | Screening question 3 for quantitative nonrandomized studies: are there complete outcome data? | Screening question 4 for quantitative nonrandomized studies: are the confounders accounted for in the design and analysis? | Screening question 5 for quantitative nonrandomized studies: during the study period, is the intervention administered (or exposure occurred) as intended? | Comments |
| --- | --- | --- | --- | --- | --- | --- | --- | --- |
| Webber et al [9], 2022 | Yes | Yes | Yes | Yes | Yes | Yes | Yes | Low risk of bias |
| Asiki et al [10], 2018 | Yes | Yes | No | Yes | Yes | Yes | No | High risk of bias: use of convenience sampling; some participants did not receive the intervention as intended |
| Atnafu [11], 2015 | Yes | Yes | Yes | Yes | Yes | No | Can’t tell | High risk of bias: the authors did not adjust for confounders (high risk of confounding bias) |
| Atnafu and Bisrat [12], 2015 | Yes | Yes | No | No | Can’t tell | No | Can’t tell | High risk of bias: no details of sample size calculation; unclear sampling method; very high nonresponse rate; no details on choices of variables and outcomes |
| Hategeka et al [13], 2019 | Yes | Yes | Yes | Yes | Yes | Yes | Yes | Low risk of bias |
| Ruton et al [14], 2018 | Yes | Yes | Yes | Yes | Yes | Yes | Yes | Low risk of bias |
| Mwendwa [15], 2018 | Yes | Yes | Yes | Yes | Yes | No | Can’t tell | High risk of bias: no adjustment of confounders; as this is a self-reported study, it is difficult to tell whether the participants received the intervention as intended |

Risk-of-bias assessment of quantitative descriptive studies using the Mixed Methods Assessment Tool.

| Study, year | Screening question 1 (for all types):are there clear research questions? | Screening question 2 (for all types): do the collected data allow to address the research questions? | Screening question 1 for quantitative descriptive studies: is the sampling strategy relevant to address the research question? | Screening question 1 for quantitative descriptive studies: is the sample representative of the target population? | Screening question 1 for quantitative descriptive studies: are the measurements appropriate? | Screening question 1 for quantitative descriptive studies: is the risk of nonresponse bias low? | Screening question 1 for quantitative descriptive studies: is the statistical analysis appropriate to answer the research question? | Comments |
| --- | --- | --- | --- | --- | --- | --- | --- | --- |
| Fulcher et al [16], 2021 | Yes | Yes | Yes | Yes | Yes | Yes | Yes | Low risk of bias |
| Ngabo et al [17], 2012 | Yes | Yes | No | No | Can’t tell | Can’t tell | Can’t tell | High risk of bias: the article lacks a sampling strategy and a description of measurements and outcomes; high risk of confounding bias |

Risk-of-bias assessment of mixed methods studies using the Mixed Methods Assessment Tool.

| Study, year | Screening question 1 (for all types): are there clear research questions? | Screening question 2 (for all types): do the collected data allow to address the research questions? | Screening question 1 for mixed methods studies: is there an adequate rationale for using a mixed methods design to address the research question? | Screening question 2 for mixed methods studies: are the different components of the study effectively integrated to answer the research question? | Screening question 3 for mixed methods studies: are the outputs of the integration of qualitative and quantitative components adequately interpreted? | Screening question 4 for mixed methods studies: are divergences and inconsistencies between quantitative and qualitative results adequately addressed? | Screening question 5 for mixed methods studies: do the different components of the study adhere to the quality criteria of each tradition of the methods involved? | Comments |
| --- | --- | --- | --- | --- | --- | --- | --- | --- |
| Webber et al [18], 2019 | Yes | Yes | Yes | Yes | No | No | No | High risk of bias: limited discussion on quantitative results in light of the qualitative results |
| Boene et al [19], 2021 | Yes | Yes | Yes | Yes | Yes | Yes | No | High risk of bias: some concern (eg, criteria for selection not discussed and limited discussion of qualitative methods and results) |
| Nigussie et al [20], 2021 | Yes | Yes | Yes | Yes | Yes | Can’t tell | No | High risk of bias: limited qualitative results and no descriptions of divergences; limited information on qualitative and quantitative methods |
| Mushamiri et al [21], 2015 | Yes | Yes | Yes | Yes | Yes | Yes | Yes | Low risk of bias |
| Battle et al [22], 2015 | Yes | Yes | Yes | Yes | Yes | Yes | No | High risk of bias: qualitative sampling methods not adequately described |

References

1. Webber G, Chirangi B, Magatti N. Experiences of a multiple intervention trial to increase maternity care access in rural Tanzania: Focus group findings with women, nurses and community health workers. Women's health (London, England). 2020;16:1745506520969617. doi: 10.1177/1745506520969617.

2. Mangwi Ayiasi R, Atuyambe LM, Kiguli J, Garimoi Orach C, Kolsteren P, Criel B. Use of mobile phone consultations during home visits by Community Health Workers for maternal and newborn care: community experiences from Masindi and Kiryandongo districts, Uganda. BMC public health. 2015;15:560. doi: https://dx.doi.org/10.1186/s12889-015-1939-3.

3. Musabyimana A, Ruton H, Gaju E, Berhe A, Grepin KA, Ngenzi J, et al. Assessing the perspectives of users and beneficiaries of a community health worker mHealth tracking system for mothers and children in Rwanda. PloS one. 2018;13(6):e0198725. doi: 10.1371/journal.pone.0198725.

4. Mwendwa P. Assessing the fit of RapidSMS for maternal and new-born health: perspectives of community health workers in rural Rwanda. Development in Practice. 2016;26(1):38-51. doi: 10.1080/09614524.2016.1112769.

5. Sevene E, Sharma S, Munguambe K, Sacoor C, Vala A, Macuacua S, et al. Community-level interventions for pre-eclampsia (CLIP) in Mozambique: A cluster randomised controlled trial. Pregnancy hypertension. 2020;21:96-105. doi: https://dx.doi.org/10.1016/j.preghy.2020.05.006.

6. Mangwi Ayiasi R, Kolsteren P, Batwala V, Criel B, Orach CG. Effect of Village Health Team Home Visits and Mobile Phone Consultations on Maternal and Newborn Care Practices in Masindi and Kiryandongo, Uganda: A Community-Intervention Trial. PloS one. 2016;11(4):e0153051. doi: https://dx.doi.org/10.1371/journal.pone.0153051.

7. Hackett K, Lafleur C, Nyella P, Ginsburg O, Lou W, Sellen D. Impact of smartphone-assisted prenatal home visits on women's use of facility delivery: Results from a cluster-randomized trial in rural Tanzania. PloS one. 2018;13(6):e0199400. doi: https://dx.doi.org/10.1371/journal.pone.0199400.

8. Atnafu A, Otto K, Herbst CH. The role of mHealth intervention on maternal and child health service delivery: findings from a randomized controlled field trial in rural Ethiopia. mHealth. 2017;3:39. doi: https://dx.doi.org/10.21037/mhealth.2017.08.04.

9. Webber G, Chirangi B, Magatti N, Mallick R, Taljaard M. Improving health care facility birth rates in Rorya District, Tanzania: a multiple baseline trial. BMC pregnancy and childbirth. 2022;22(1):74. doi: 10.1186/s12884-022-04408-5.

10. Asiki G, Newton R, Kibirige L, Kamali A, Marions L, Smedman L. Feasibility of using smartphones by village health workers for pregnancy registration and effectiveness of mobile phone text messages on reduction of homebirths in rural Uganda. PloS one. 2018;13(6):e0198653. doi: https://dx.doi.org/10.1371/journal.pone.0198653.

11. Atnafu A. Assessment of voluntary community health workers participation and contribution in mHealth intervention. Ethiopian Journal of Health Development. 2015;29(3):154-9.

12. Atnafu A, Bisrat A. Asessment of health extension workers (HEWs) knowledge, attitude and performance in mHealth intervention in Guraghe Zone of Ethiopia: Cross-sectional study. Ethiopian Journal of Health Development. 2015;29(3):160-9.

13. Hategeka C, Ruton H, Law MR. Effect of a community health worker mHealth monitoring system on uptake of maternal and newborn health services in Rwanda. Global health research and policy. 2019;4:8. doi: https://dx.doi.org/10.1186/s41256-019-0098-y.

14. Ruton H, Musabyimana A, Gaju E, Berhe A, Grepin KA, Ngenzi J, et al. The impact of an mHealth monitoring system on health care utilization by mothers and children: an evaluation using routine health information in Rwanda. Health policy and planning. 2018;33(8):920-7. doi: https://dx.doi.org/10.1093/heapol/czy066.

15. Mwendwa P. What encourages community health workers to use mobile technologies for health interventions? Emerging lessons from rural Rwanda. Development Policy Review. 2018;36(1):111-29. doi: 10.1111/dpr.12275.

16. Fulcher IR, Nelson AR, Tibaijuka JI, Seif SS, Lilienfeld S, Abdalla OA, et al. Improving health facility delivery rates in Zanzibar, Tanzania through a large-scale digital community health volunteer programme: a process evaluation. Health policy and planning. 2021;35(10):1-11. doi: https://dx.doi.org/10.1093/heapol/czaa068.

17. Ngabo F, Nguimfack J, Nwaigwe F, Mugeni C, Muhoza D, Wilson DR, et al. Designing and Implementing an Innovative SMS-based alert system (RapidSMS-MCH) to monitor pregnancy and reduce maternal and child deaths in Rwanda. The Pan African medical journal. 2012;13:31.

18. Webber GC, Chirangi BM, Magatti NJ. Challenges and Successes of Distributing Birth Kits with Misoprostol to Reduce Maternal Mortality in Rural Tanzania. African journal of reproductive health. 2019;23(3):68-78. doi: 10.29063/ajrh2019/v23i3.7.

19. Boene H, Vala A, Kinshella M-LW, La M, Sharma S, Vidler M, et al. Implementation of the PIERS on the Move mHealth Application From the Perspective of Community Health Workers and Nurses in Rural Mozambique. Frontiers in global women's health. 2021;2:659582. doi: https://dx.doi.org/10.3389/fgwh.2021.659582.

20. Nigussie ZY, Zemicheal NF, Tiruneh GT, Bayou YT, Teklu GA, Kibret ES, et al. Using mHealth to Improve Timeliness and Quality of Maternal and Newborn Health in the Primary Health Care System in Ethiopia. Global health, science and practice. 2021;9(3):668-81. doi: https://dx.doi.org/10.9745/GHSP-D-20-00685.

21. Mushamiri I, Luo C, Iiams-Hauser C, Ben Amor Y. Evaluation of the impact of a mobile health system on adherence to antenatal and postnatal care and prevention of mother-to-child transmission of HIV programs in Kenya. BMC public health. 2015;15:102. doi: https://dx.doi.org/10.1186/s12889-015-1358-5.

22. Battle JD, Farrow L, Tibaijuka J, Mitchell M. mHealth for Safer Deliveries: A mixed methods evaluation of the effect of an integrated mobile health intervention on maternal care utilization. Healthc (Amst). 2015 Dec;3(4):180-4. PMID: 26699340. doi: 10.1016/j.hjdsi.2015.10.011.
